# Predicting the in vivo pulmonary toxicity induced by acute exposure to poorly soluble nanomaterials by using advanced in vitro methods

**DOI:** 10.1186/s12989-018-0260-6

**Published:** 2018-06-04

**Authors:** Thomas Loret, Françoise Rogerieux, Bénédicte Trouiller, Anne Braun, Christophe Egles, Ghislaine Lacroix

**Affiliations:** 1 Institut National de l’Environnement Industriel et des Risques (INERIS), (DRC/VIVA/TOXI), Parc Technologique ALATA - BP 2, F-60550 Verneuil-en-Halatte, France; 2 Université de Technologie de Compiègne (UTC), Laboratoire BioMécanique et BioIngénierie (BMBI), UMR CNRS 7338, 60205 Compiègne, France; 3 Department of Biomedical Engineering, Tufts University, Medford, MA USA

**Keywords:** Poorly soluble nanomaterials, Acute exposure, Pulmonary toxicity, Alternative toxicity testing, Air-liquid interface, In vivo - in vitro comparison

## Abstract

**Background:**

Animal models remain at that time a reference tool to predict potential pulmonary adverse effects of nanomaterials in humans. However, in a context of reduction of the number of animals used in experimentation, there is a need for reliable alternatives. In vitro models using lung cells represent relevant alternatives to assess potential nanomaterial acute toxicity by inhalation, particularly since advanced in vitro methods and models have been developed. Nevertheless, the ability of in vitro experiments to replace animal experimentation for predicting potential acute pulmonary toxicity in human still needs to be carefully assessed.

The aim of the study was to evaluate the differences existing between the in vivo and the in vitro approaches for the prediction of nanomaterial toxicity and to find advanced methods to enhance in vitro predictivity. For this purpose, rats or pneumocytes in co-culture with macrophages were exposed to the same poorly soluble and poorly toxic TiO_2_ and CeO_2_ nanomaterials, by the respiratory route in vivo or using more or less advanced methodologies in vitro. After 24 h of exposure, biological responses were assessed focusing on pro-inflammatory effects and quantitative comparisons were performed between the in vivo and in vitro methods, using compatible dose metrics.

**Results:**

For each dose metric used (mass/alveolar surface or mass/macrophage), we observed that the most realistic in vitro exposure method, the air-liquid interface method, was the most predictive of in vivo effects regarding biological activation levels. We also noted less differences between in vivo and in vitro results when doses were normalized by the number of macrophages rather than by the alveolar surface. Lastly, although we observed similarities in the nanomaterial ranking using in vivo and in vitro approaches, the quality of the data-set was insufficient to provide clear ranking comparisons.

**Conclusions:**

We showed that advanced methods could be used to enhance in vitro experiments ability to predict potential acute pulmonary toxicity in vivo. Moreover, we showed that the timing of the dose delivery could be controlled to enhance the predictivity. Further studies should be necessary to assess if air-liquid interface provide more reliable ranking of nanomaterials than submerged methods.

**Electronic supplementary material:**

The online version of this article (10.1186/s12989-018-0260-6) contains supplementary material, which is available to authorized users.

## Background

Inhalation is an important exposure route for many metallic and poorly soluble nanomaterials (NMs) [1], including TiO_2_ or CeO_2_, which are among the most commonly used in nanotechnologies [2]. To assess the pulmonary toxicity of these NMs after acute exposure, in vivo assays using animal models remain the most reliable approach to predict potential adverse effects in humans [3], because of similar levels of complexity. Nevertheless, considering the high number of NMs used and their physico-chemical diversity, it seems difficult, for ethical and financial reasons, to rely on animal experimentation only. It is therefore necessary to find reliable methods that can be used as alternatives to in vivo models in this context.

In vitro studies using lung cells represent an inexpensive and easy-to-use alternative to assess pulmonary acute toxicity after exposure to NMs [4]. Usually in vitro, the cells are exposed in submerged conditions to suspensions of NMs for 24 h. However, these simplistic experimental conditions do not accurately mimic the interactions between particles and lungs in the human body [5]. This may lead to different biological responses between in vivo and in vitro approaches. Recently, many progresses have been made to simulate in vitro the cell–particle interactions occurring in the lungs in vivo. Importantly, advanced cellular models including co-cultures or 3D-cultures [6] and physiological exposure methods, including systems allowing exposure of cells at the air-liquid interface (ALI) to aerosols of NMs [7], have been developed. These new methodologies could help to predict more reliably the pulmonary effects observed in vivo.

Comparisons of NMs toxicity between in vivo and in vitro approaches were performed in several studies to assess if similar toxicity patterns could be found. Qualitative *vivo-vitro* comparisons were performed. In their study, Sayes et al. [8] compared cytotoxic and inflammatory responses, between rats exposed in vivo by intratracheal instillation and alveolar epithelial cells and macrophages exposed in vitro in submerged conditions to silicium and ZnO NMs. The authors didn’t observe correlations between the in vitro and in vivo results. Nevertheless, Rushton et al. [9] highlighted that better *vivo*-*vitro* correlations could be obtained when the toxicological responses were normalized by the NM surface areas. In this work, the authors normalized the data published by Sayes et al. by the surface area of the NMs and showed that the NMs used could be ranked similarly in vivo and in vitro in function of their toxicity. Recently, it has also been shown that advanced comparisons could be performed by using similar dose metrics between in vivo and in vitro approaches. For example, Kim et al. [10] performed quantitative comparisons between mice exposed in vivo by oropharyngeal aspiration and lung slices or alveolar macrophages exposed in vitro to suspensions of TiO_2_ and CeO_2_ NMs. For some NMs, they showed pro-inflammatory effects at similar doses in vivo and in vitro when the doses were expressed in mass of NM per surface unit, both in vivo and in vitro. Teeguarden et al. [11] compared the pulmonary toxicity between mice exposed in vivo by inhalation and alveolar epithelial cells or macrophages exposed in vitro in submerged conditions to iron oxide NMs. They showed inflammatory effects at lower doses in vivo compared to in vitro when the doses were expressed in μg/cm^2^ and better similarity when the doses were expressed in mass of NM per number of macrophages. Donaldson et al. [12] showed good correlations between pro-inflamatory responses in vivo in rats (neutrophil influx) and in vitro (IL-8 expression) in A549 cells when the doses expressed in μg/cm^2^ where normalized by NM surface areas. Nevertheless, only few in vitro experiments performed in submerged conditions were compared to in vivo experiments and it remains unclear whether better prediction could be obtained by using more advanced in vitro methods, like ALI exposures.

In this context, the aim of our study was to assess the ability of several in vitro methods, more or less advanced, to predict the adverse effects observed in vivo after exposure to poorly toxic and poorly soluble metallic NMs. The perspective is to promote reliable alternative methodologies to predict the potential inhalation toxicity of NMs in humans. For this purpose, in vivo and in vitro experiments were performed using the same TiO_2_ and CeO_2_ NMs. In vivo, rats were exposed to the NMs by intratracheal instillation and then sacrificed after 24 h of exposure. In vitro, alveolar epithelial cells in co-culture with macrophages were exposed for 24 h at the ALI to aerosols or in submerged conditions to suspensions of NMs. Moreover, different deposition kinetics were tested. The results of the in vitro study were published previously by our team [13]. In this paper we showed toxic effects at lower doses when cells were exposed at the ALI to aerosols of NMs compared to exposure to suspensions. We also showed the relevance of timing consideration for the dose delivery when assessing poorly soluble NM toxicity in vitro. Both in vivo and in vitro, cytotoxic, inflammatory and oxidative stress responses were assessed after 24 h of exposure and qualitative and quantitative comparisons were performed. To perform in vivo *-* in vitro comparisons, common dose metrics were selected between in vivo and in vitro methods and normalizations were performed.

## Results

The ability of several in vitro methods (ALI and submerged) to predict potential adverse effects in vivo in lungs, after exposure to poorly toxic and poorly soluble metallic NMs, was assessed in this study.

For this purpose, we performed in vivo and in vitro experiments using the same TiO_2_ (NMs 105, 101 and 100) and CeO_2_ (NM212) NMs. The physico-chemical characteristics of the four NMs were characterized in exposure media (Table 1). Furthermore, the number size distributions and densities of NMs in suspensions (for exposure of cells in submerged conditions in vitro or rats by intratracheal instillation in vivo) and in aerosols (for exposure of cells at the ALI) were assessed. Surprisingly, similar results were observed between NM suspended in water and in culture medium [13]. Number size distributions and densities determined in exposure media were then used to calculate the mean surface area of NM agglomerates in suspensions and in aerosols (for ALI exposures only). Based on our previous electron microscopy observations [13], agglomerates were considered spherical for the calculation. The mean surface area calculated in exposure media was then used for *vivo-vitro* comparisons.

**Table 1:** Physico-chemical properties of TiO_2_ (NMs 105, 101, 100) and CeO_2_ (NM212) nanomaterials in exposure media

	Critallinity	Coating	Primary particle size (nm)	Primary density (g/cm^3^)	Primarysurface area, BET (m^2^/g)	Mean size in exposure media (nm)		Mean density in exposure media (g/cm^3^)		Mean surface area in exposure media (m^2^/g)	
						Susp	Aero	Susp	Aero	Susp	Aero
NM105	80% anatase / 20% rutile	No	21	4.2	46.1	318	240	1.4	0.7	13.5	37.7
NM101	Anatase	Hydrophobic	8	3.9	316	567	80	1.6	0.9	6.7	83.3
NM100	Anatase	No	100	3.9	10	286	320	1.8	0.6	11.7	31.3
NM212	Cubic cerionite	No	29	7.2	27	233	200	2.1	1.1	12.5	27.3

*Susp* Suspension, *Aero* Aerosol

**Fig. 1:**
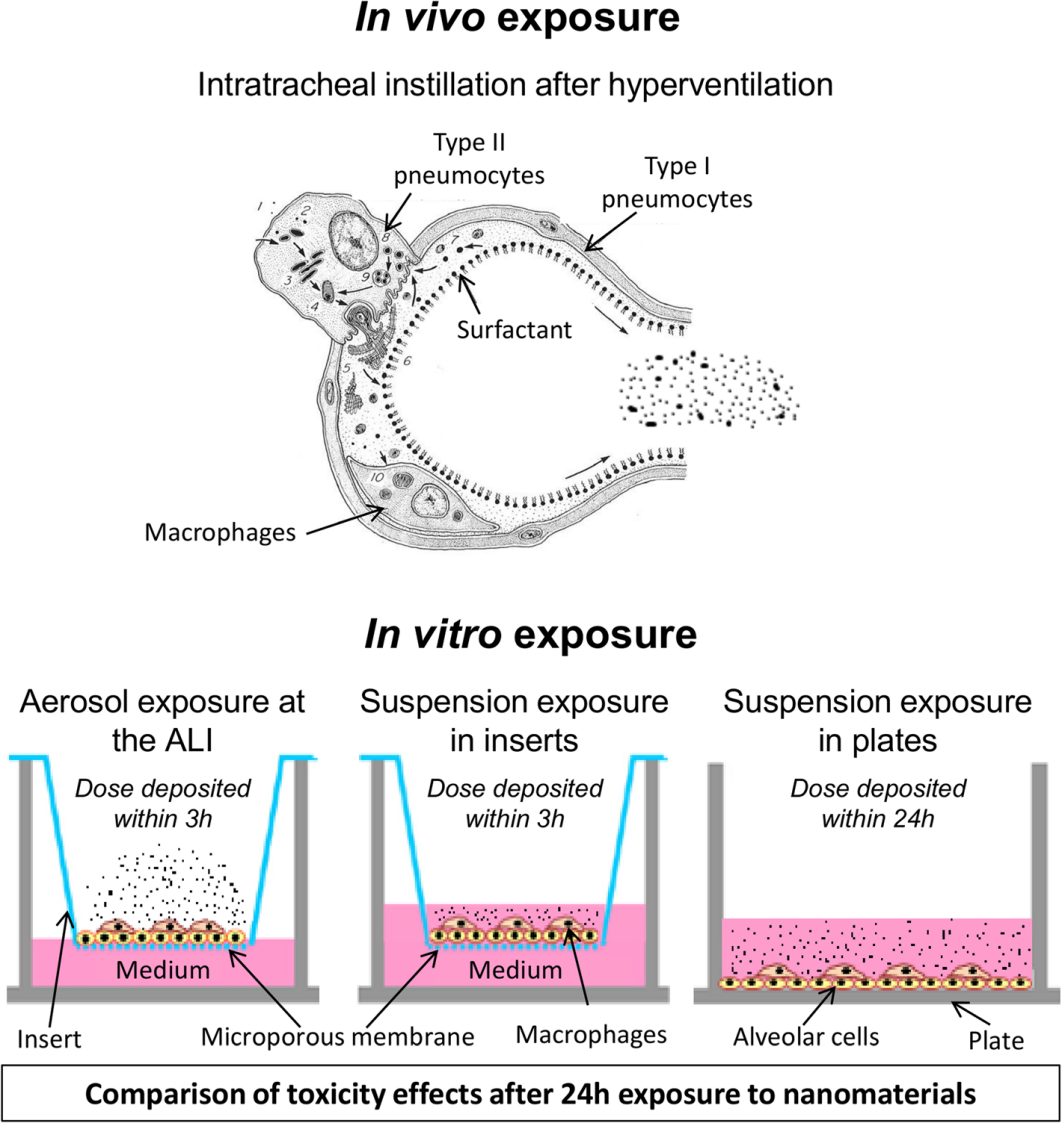
Experimental conditions used for the in vivo/in vitro comparisons (adapted from [13]). In vitro and in vivo experiments were performed using the same TiO_2_ (NM105, NM101, NM100) and CeO_2_ (NM212) NMs. In vitro, alveolar epithelial cells in co-culture with macrophages were exposed for 24 h at the air-liquid interface (ALI) to aerosols or in submerged conditions to suspensions of NMs. Different deposition kinetics were tested. At the ALI the NM deposition via aerosol was maintained for 3 h. The cells were then kept at the incubator for the remaining 21 h (3 h + 21 h). In submerged conditions, two deposition kinetics were used. In inserts, the deposition was maintained for 3 h. After 3 h, NM suspensions were replaced by fresh medium and the cells were then kept a the incubator for the remaining 21 h (3 h + 21 h) with the NMs deposited on their surface. In plates, classic exposure conditions were used and NM depositions were maintained for 24 h. In vivo, rats were exposed by intratracheal instillation with NM suspension and the NM were deposited almost instantly into the lungs. After 24 h of exposure, the biological activity was assessed, focusing more particularly on pro-inflammatory markers, including cytokine secretions and neutrophil influx (in vivo only)

The major innovation of this study was to compare NM toxicities between in vivo and in vitro approaches, using several more or less advanced in vitro methods and testing different timings of the dose delivery in vitro. The Fig. 1, which was adapted from our previous published paper [13] to take into account our new in vivo experiments, is presented here and proposes an overview of the study design. For the study, we focused on the doses deposited into the lungs (in vivo) or on cells (in vitro) because we postulated that metallic and poorly soluble NMs exert their toxicity by direct contact with the cells.

In vivo, rats were exposed to NMs by intratracheal instillation and sacrificed after 24 h of exposure. In vitro, alveolar epithelial cells in co-culture with macrophages were exposed for 24 h at the ALI to aerosols or in submerged conditions to the NMs. At the ALI, the cells were exposed to aerosols of NMs for 3 h, meaning that the final deposited dose was reached within 3 h. The cells were then kept in the incubator for the remaining 21 h at the ALI with the NMs deposited on their surface. In submerged conditions, we used two different timings of the dose delivery. Nevertheless, as shown in our previous paper [13], NMs concentration in suspensions were adjusted to obtain similar deposited doses (Additional file 1: Table S1). First, cells were exposed to suspensions in inserts and the NM deposition was maintained for 3 h. After 3 h of exposure, the deposition was stopped and the cells were kept during the remaining 21 h in submerged condition in the incubator. Secondly, cells were exposed in plates to suspensions of NMs for 24 h. In that situation, the NM deposition was maintained for the whole exposure time, meaning that the final deposited dose was reached within 24 h. After 24 h of exposure, inflammation, cytotoxicity and oxidative stress were assessed. Lowest Observed Adverse Effects Levels (LOAELs) and critical effect dose intervals were then determined, using first significant effects measured or benchmark dose response modeling, respectively. Focusing on these LOAELs and critical effect dose intervals, quantitative and qualitative comparisons were performed between in vivo and in vitro results. With dose intervals, contrary to with LOAELs, dose-response curves were taken into account and uncertainty was included in the data. Comparisons were performed with LOAELs and dose intervals to assess if similar conclusions could be made using the two criteria of effect dose. For these comparisons, normalizations were performed to have common dose metrics between in vivo and in vitro approaches. NM surface areas were also considered for ranking comparisons.

### Pro-inflammatory responses in vivo and in vitro

In vivo and in vitro, the biological responses were assessed after 24 h of exposure to three TiO_2_ (NMs 105, 101, 100) and one CeO_2_ (NM212) NMs.

**Fig. 2:**
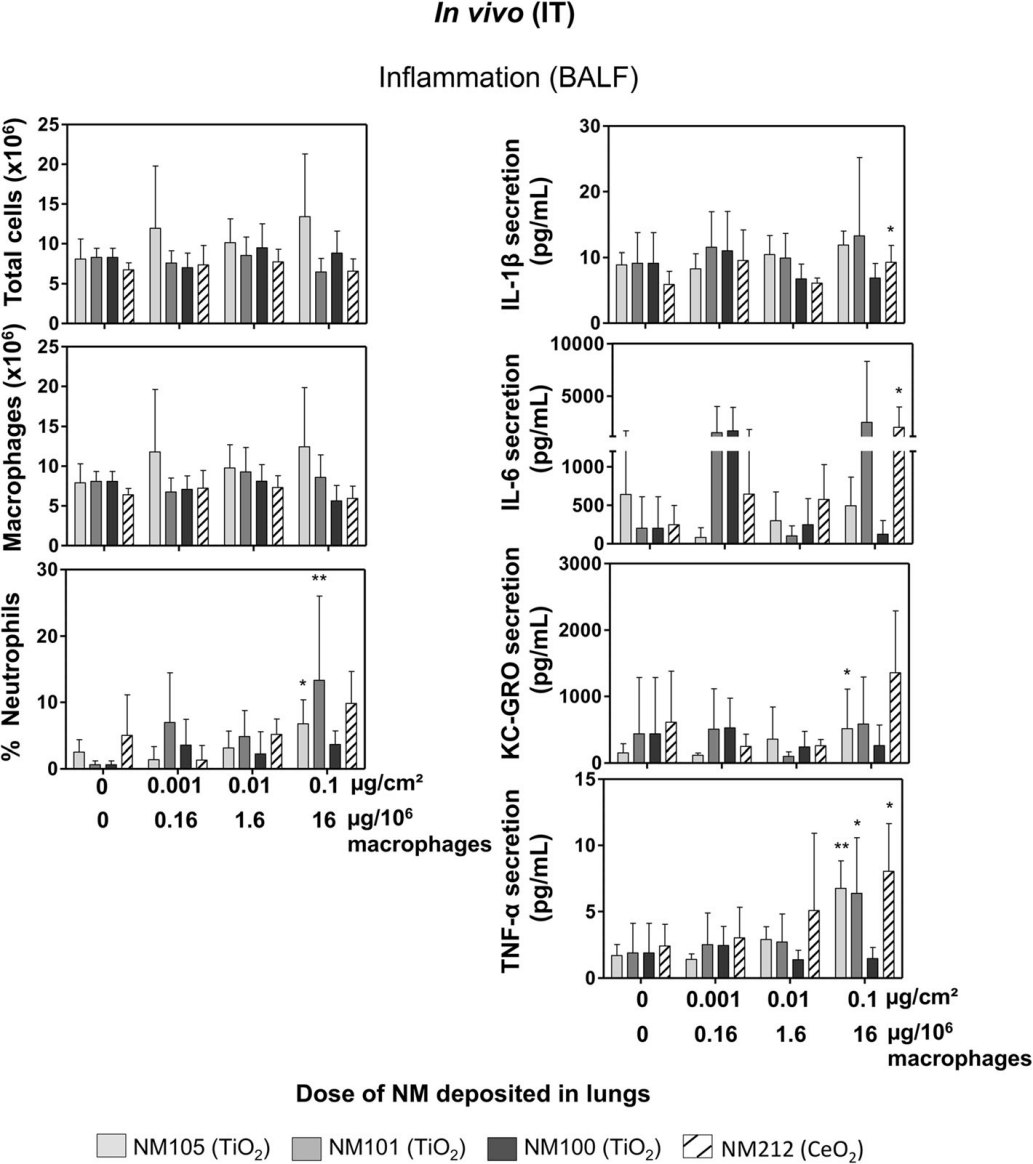
Cytology and cytokines/chemokine levels in bronchoalveolar lavage fluids 24 h after instillation with the NMs. Rats were instilled after hyperventilation with suspensions of TiO_2_ (NM105, NM101, NM100) and CeO_2_ (NM212). After sacrifice, bronchoalveolar lavages were performed using PBS. The bronchoalveolar lavage fluids were recovered and centrifuged to separate cells from supernatant. For cytology analysis, the cells were resuspended in RMPI medium and then seeded on slides at 300000 cells/spots using a cytospin and then fixated and coloured in May-Grunwald Giemsa. The percentage of different cell types in BALF was determined using optical microscopy. For cytokine/chemokine analysis, supernatants were dosed using ELISA multiplex to determine IL-1β, IL-6, KC-GRO and TNF-α levels. Data represent the mean ± SD of six animals. Kruskal-Wallis test followed by Dunn’s post-hoc test were performed to compare treated groups to controls (*p < 0.05; **p < 0.01; ***p < 0.001)

In vivo, pro-inflammatory effects (neutrophil influx and levels of the pro-inflammatory mediators IL-1β, IL-6, KC-GRO and TNF-α) were assessed in bronchoalveolar lavage fluids (BALF) of rats exposed by intratracheal instillation (IT) to the NMs (around 4.5 mL recovered for each sample). We observed significant effects with TiO_2_ NMs 105 and 101 and CeO_2_ NM212, but not with TiO_2_ NM100. Significant pro-inflammatory effects were noted at the maximum dose tested: 400 μg/lungs, corresponding to around 0.1 μg/cm^2^ or 20 μg/10^6^ macrophages after normalization by the alveolar surface (4000 cm^2^) or the number of alveolar macrophages (25 million), respectively (Fig. 2). After exposure to TiO_2_NMs 105 and 101, this was characterized by a significant neutrophil influx in BALF supernatants, associated with increased concentrations of TNF-α for the NMs 105 and 101 and KC-GRO for NM105 only. We also noted significant increases in IL-1β, IL-6 and TNF-α secretion with NM212, although no significant neutrophil influx was detected. The absence of significant neutrophil influx with NM212 may have been due to a high variability in the control sample. No significant increases in neutrophils or cytokines were noted for TiO_2_ NM100. Moreover, for all the NMs tested, we did not observe any significant changes in macrophages or total cell numbers.

Based on the significant responses detected in vivo, lowest observed adverse effects levels (LOAELs) were determined for pro-inflammatory effects with NMs 105, 101 and 212, but not with NM100. These LOAELs were used in the present study to compare in vivo and in vitro results. We also used benchmark dose-response modeling to determine critical effect doses for a 20% increase of cytokine/chemokine response with an interval of doses corresponding to a 90% confidence, to compare in vivo and in vitro results.

In vitro, pro-inflammatory responses were assessed after 24 h of exposure by evaluating the levels of pro-inflammatory mediators IL-1β, IL-6, IL-8 and TNF-α in cell supernatants. After ALI exposure to aerosols of NM in inserts, cytokine levels were only measured in the basolateral compartment (containing 2 mL of culture medium) as the cells were maintained at the ALI for the 3 h of exposure to the aerosols and for the remaining 21 h into the incubator with the NMs deposited on their surface. After exposure in submerged conditions to suspensions in inserts, using the similar dose rate timing of the dose delivery than at the ALI (3 h), cytokine levels were assessed both in the apical and basolateral compartments of the inserts (containing 1 and 2 mL of culture medium, respectively). In submerged conditions in plates, which represents the classic exposure conditions usually used in vitro, the NM deposition was maintained for the 24 h of exposure and cytokine levels were exclusively measured on the apical side of the cells (containing 0.5 mL of culture medium) due to the absence of a basolateral compartment.

Briefly, as demonstrated in our previous in vitro study [13], we observed significant pro-inflammatory responses at the ALI with all tested NMs. We also observed effects in submerged conditions in inserts and in plates, but mainly with NMs 105 and 101. A compilation of the pro-inflammatory results published in our previous study [13] is available in the Additional files of the present paper (Additional file 1: Figure S1). According to the first significant pro-inflammatory responses detected, LOAELs were determined for each assay performed (Additional file 1: Table S2). For all NMs, the LOAELs were determined at lower doses at the ALI compared to submerged conditions in inserts and also at lower doses when the final dose was deposited within 3 h rather than within 24 h. In the present study, benchmark dose-response modeling was also used with the in vitro data to determine an interval of dose for a 20% increase of cytokine/chemokine response with a 90% confidence, to compare in vivo and in vitro results.

### Cytotoxicity and oxidative stress effects in vivo and in vitro

Cytotoxicity and oxidative stress responses were also assessed, both in vivo and in vitro. In vivo, LDH levels were evaluated in BALF supernatants and Reactive Oxygen Species (ROS) levels were measured in BALF cells. Although significant pro-inflammatory responses were noted, we did not observe any significant cytotoxic or oxidative stress effects after 24 h of exposure to the NMs (Table 2 and Additional file 1: Figure S2). In vitro, cytotoxicity was assessed by using the alamar blue test and by measuring LDH levels in cell supernatants. ROS levels were measured in cells as marker of oxidative stress. We observed few significant cytotoxicity and oxidative responses at the ALI (and only with the NMs 105 and 101). LOAELs were also determined for cytotoxicity and oxidative stress in submerged conditions and more particularly in inserts (Table 2). As described in our previous article [13], it was not possible to perform clear quantitative comparisons between the different in vitro exposure method using these two parameters because too little significant cytotoxicity and oxidative stress effects were detected in vitro. However, it could not be excluded that less cytotoxicity and oxidative stress effects were observed compared to pro-inflammatory effects, both in vivo and in vitro, because of a lack of sensitivity of the assays performed.

**Table 2:**
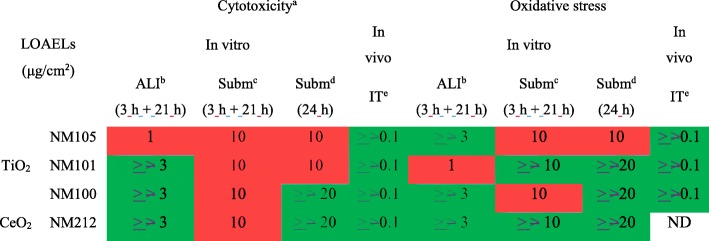
LOAELs (μg/cm^2^) for cytotoxicity and oxidative stress effects determined after 24 h of exposure

^a^LOAELs indicated represent significant cytotoxicity > 5%

Significant effects allowing the determination of a LOAEL

No significant adverse effects observed

^b^Doses tested at the ALI: 0.1, 1, 3 μg/cm^2^

^c^Doses tested in submerged conditions in inserts: 1, 3, 10 μg/cm^2^

^d^Doses tested in submerged conditions in plates: 1, 3, 10, 20 μg/cm^2^

^e^Doses tested in vivo: 0.001, 0.01, 0.1 μg/cm^2^

### *Vivo-vitro* comparisons using the inflammation results

As described previously, inflammation was the most sensitive marker of biological responses at 24 h in our study, both in vivo and in vitro. For this reason, we focused on the pro-inflammatory responses to perform *vivo-vitro* comparisons. To perform quantitative comparisons, the LOAELs determined in vivo and in vitro for the first significant pro-inflammatory responses observed were first used. Dose-response comparisons were then performed using dose intervals determined by benchmark modeling. For dose intervals calculation, we determined a critical effect dose corresponding to a 20% increase of pro-inflammatory mediator levels compared to non-exposed controls and the Benchmark Dose Lower confidence limit (BMDL) and the Benchmark Dose Upper confidence limit (BMDU) of the interval for a 90% confidence. This was performed for each pro-inflammatory mediator, each exposure method and each NM used. Examples of benchmark dose-response modeling for the calculation of critical effect doses and dose intervals are shown in the Additional file 1: Figure S3. For each NM and each exposure method, we then calculated the median value of the BMDL and the median value of the BMDU for the four pro-inflammatory mediators (IL-1β, IL-6, IL-8/KC-GRO, TNF-α), to determine a median dose interval for general pro-inflammatory response, as shown in the Table 3. We calculated the median dose interval of the four cytokines because similar results could be observed in our study when pooling the results of the four cytokines and when comparing dose intervals of each cytokine one by one. We believe that comparisons performed in our study were easier to follow when a general pro-inflammatory response was used instead of comparing the dose intervals of each cytokine one by one. With dose intervals, contrary to with LOAELs, dose-response curves were taken into account and uncertainty was included in the data. Comparisons were performed with LOAELs and dose intervals to assess if similar conclusions could be made using the two criteria of dose. For the comparisons, LOAELs and dose intervals were expressed using different dose metrics which were compatible between in vivo and in vitro methods.

**Table 3:** Dose intervals (in μg/cm^2^) determined for each NM and each methodology

	Cytokines	NM105			NM101			NM100			NM212		
		Dose interval			Dose interval			Dose interval			Dose interval		
		BMDL	BMDU	Median	BMDL	BMDU	Median	BMDL	BMDU	Median	BMDL	BMDU	Median
In vitro, suspension (24 h)	IL-1β	0.10	11.90	4.63–11.20	0.53	2.85	2.82–6.36	10.68	32.59	10.96–26.19	0.06	10.26	0.430–14.168
	IL-6	4.50	11.11		4.86	10.47		ND	ND		ND	ND	
	IL-8	ND	ND		ND	ND		ND	ND		ND	ND	
	TNF-α	4.76	11.20		2.82	6.36		11.25	19.80		0.80	18.08	
In vitro, suspension (3 h + 21 h)	IL-1β	0.43	2.25	0.26–1.51	0.60	6.61	0.80–6.19	3.47	53.23	3.47–55.64	3.47	54.81	3.37–52.50
	IL-6	0.20	1.36		1.28	8.17		ND	ND		2.30	50.25	
	IL-8	0.11	1.11		0.84	3.73		3.35	58.05		3.42	9.64	
	TNF-α	0.31	1.66		0.76	5.78		3.83	52.87		3.31	54.75	
ALI (3 h + 21 h)	IL-1β	0.051	0.80	0.061–0.82	0.061	0.74	0.099–0.80	0.006	0.91	0.045–0.90	0.63	2.61	0.88–2.71
	IL-6	0.037	0.81		0.089	0.77		0.012	0.88		0.88	2.71	
	IL-8	0.078	0.83		1.13	11.87		0.078	0.90		ND	ND	
	TNF-α	0.070	0.82		0.11	0.84		0.24	0.97		ND	ND	
In vivo	IL-1β	0.0011	0.067	0.0007–0.075	ND	ND	0.0022–0.084	ND	ND	ND	ND	ND	0.0000–0.074
	IL-6	0.00069	0.13		0.0044	0.086		ND	ND		0.000	0.019	
	IL-8	0.00019	0.084		ND	ND		ND	ND		0.0033	0.082	
	TNF-α	0.00075	0.0091		0.000	0.082		ND	ND		0.000	0.074	

*BMDL* Benchmark Dose Lower confidence limit, *BMDU* Benchmark Dose Upper confidence limit. Median: Median BMDL and BMDU values calculated by pooling the four cytokines, to have a dose-interval for a general pro-inflammatory response

#### Selection of relevant dose metrics

**Fig. 3:**
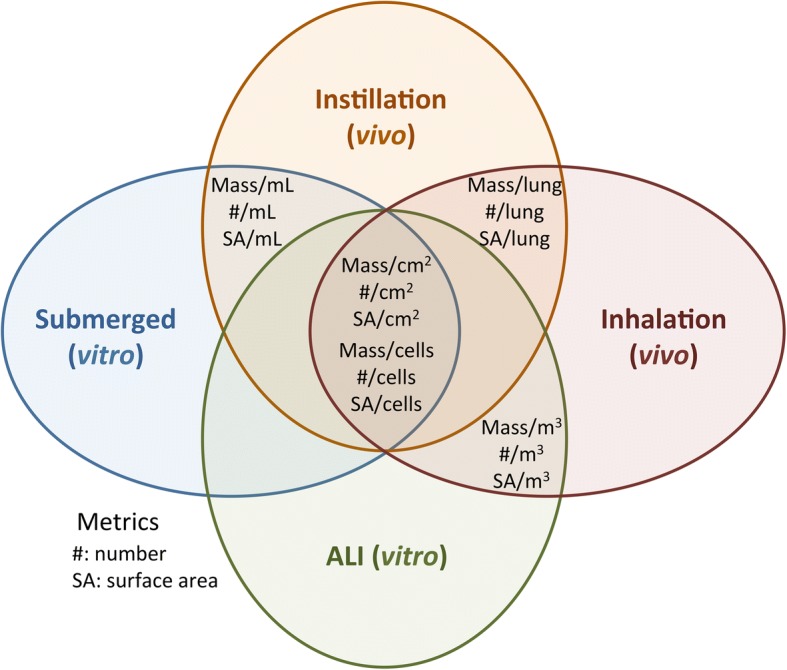
Compatibility of the different dose metrics between in vivo and in vitro approaches. In order to compare in vitro and in vivo conditions it is important to use common dose metrics. The doses are often expressed as concentrations, including mass/volume of liquid in vitro in submerged conditions and mass/volume of air in vivo in inhalation studies. However, these metrics cannot be used within the different in vivo (inhalation or instillation) and in vitro (ALI or submerged) methodologies. Moreover, using concentrations in mass/volume does not take into account the real contact between the NMs and the cells or tissues. Thus it does not seem appropriate to use such dose metrics for in vivo-in vitro comparisons; more particularly for poorly soluble NMs as their toxicity is attributable to their surface reactivity. In vivo, the total mass of NMs administered per lungs, animal or mass is often used as dose metric. This dose metric takes into account the deposition in the overall organ, but cannot be used in vitro. Nevertheless, common dose metrics can be used by normalizing the mass deposited on cells in vitro or into the lungs in vivo by the surface of the tissues or by the number of cells. Doses expressed in mass can also be normalized NM surface areas, that has been shown to be the most effective dose metric for acute NM toxicity in the lung

Common dose metrics that could be used with all exposure methods (submerged, ALI, instillation or inhalation) were selected, as shown in Fig. 3, to compare NM toxicity between in vivo and in vitro approaches. To generate common dose metrics, we normalized the deposited masses by the alveolar surface or by the macrophage number [14]. These two normalizations were performed as they take into account the direct contact between the NMs and the tissues, that was shown to be the main cause of toxicity for poorly soluble NMs [15–17]. To express the metric in mass per alveolar surface unit, masses deposited into the lungs or on the cells were divided by the total alveolar surface in vivo (4000 cm^2^) [18, 19] or by the surface of the cell layer in vitro (4,67 cm^2^ in inserts and 2 cm^2^ in plates), respectively. To express the metric in mass per macrophage number, the mass of NM in lungs (in vivo) or the deposited mass per cm^2^ (in vitro) were divided by the total number of alveolar macrophages in vivo (around 25 million) [18, 20] or in vitro (60,000 or 25,000/cm^2^ in inserts or in plates, respectively) [13]. The doses expressed in mass/macrophages were also normalized by the surface area for each NM. This normalization was performed because it was shown that the surface area was the most effective dose metric to explain acute NM toxicity in the lung [15–17]. For that, doses expressed in mass/macrophages were multiplied by NM surface areas, calculated using NM primary characteristics in powders (BET method) or mean sizes and densities in exposure media. Based on our previous observations, NMs were assumed to be spherical for surface area calculations in exposure media [13]. Nevertheless, it was not possible to ensure that NM agglomerates were strictly spherical and relative uncertainties remain regarding the mean surface area calculated in exposure media.

Doses could also be expressed in number of NMs per surface area or per cell. Nevertheless, these metrics were not chosen due to the difficulty to characterize NM size distributions in the lungs. Moreover, the number metric was not shown to be more relevant than the mass metric when assessing NM toxicity [16].

#### Comparisons in mass/alveolar surface

**Fig. 4:**
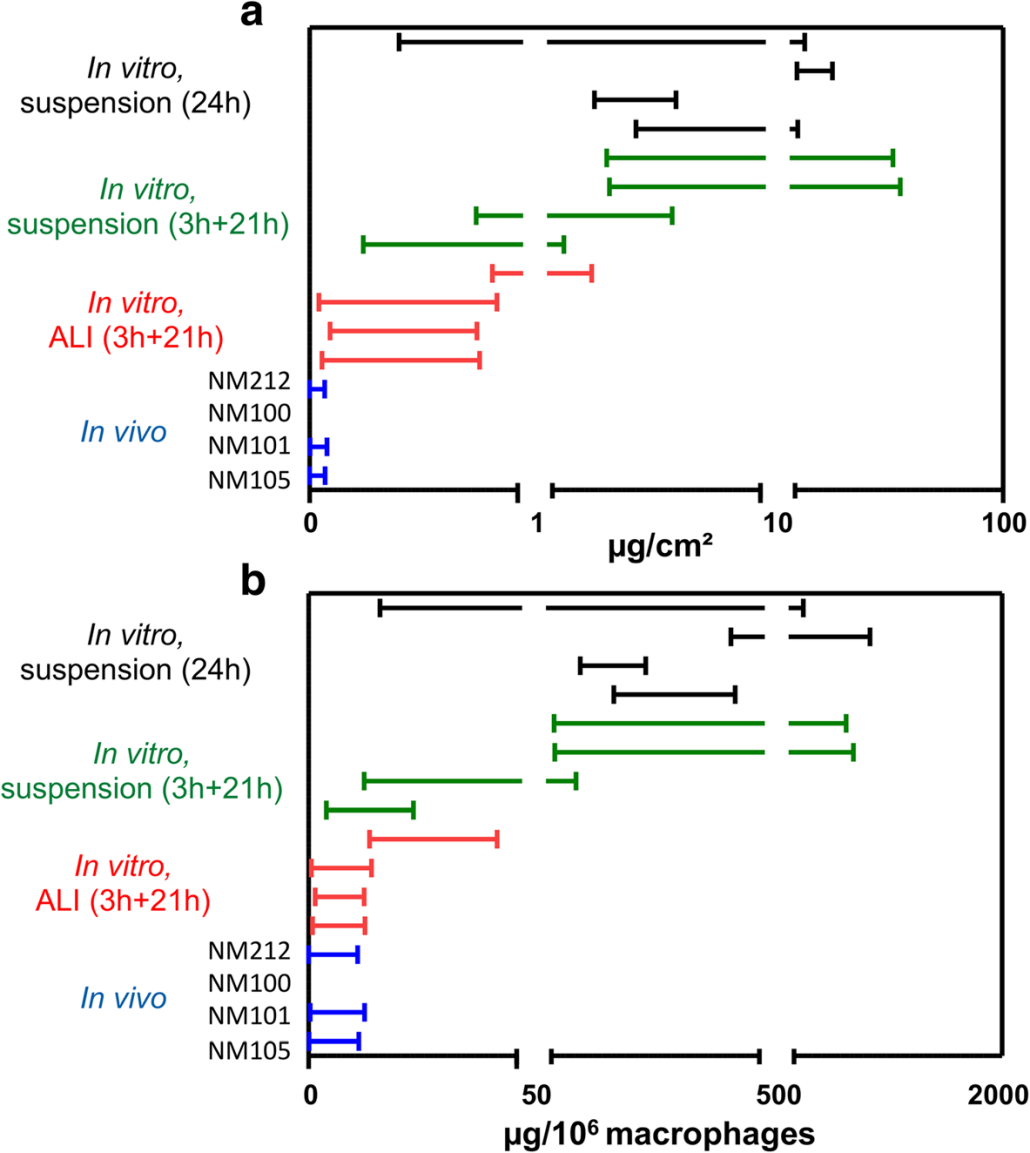
Dose intervals calculated for a 20% increase in inflammation markers in function of methodologies used. Comparisons of dose intervals were performed between the in vivo and in vitro methods used. The comparisons were performed using two dose metrics: the mass/alveolar surface (**a**) or the mass/macrophages (**b**). In vitro and in vivo experiments were performed using the same TiO_2_ (NM105, NM101, NM100) and CeO_2_ (NM212) NMs. In vitro, alveolar epithelial cells in co-culture with macrophages were exposed for 24 h at the air-liquid interface (ALI) to aerosols or in submerged conditions to suspensions of NMs. Different deposition kinetics were tested. At the ALI the NM deposition via aerosol was maintained for 3 h. The cells were then kept at the incubator for the remaining 21 h (3 h + 21 h). In submerged conditions, two deposition kinetics were used. In inserts, the deposition was maintained for 3 h. After 3 h, NM suspensions were replaced by fresh medium and the cells were then kept a the incubator for the remaining 21 h (3 h + 21 h) with the NMs deposited on their surface. In plates, classic exposure conditions were used and NM depositions were maintained for 24 h. In vivo, rats were exposed by intratracheal instillation with NM suspensions and the NMs were deposited almost instantly into the lungs. After 24 h of exposure, the biological activity was assessed, focusing more particularly on pro-inflammatory mediators. For each exposure method and for each NM, benchmark dose-response modeling was used to estimate the critical dose related to a 20% increase of pro-inflammatory mediator level and the lowest (BMDL) and the highest (BMDU) dose of the interval corresponding to confidence interval of 90%. A median dose intervals was then calculated by pooling the dose intervals of the four cytokine to have a general pro-inflammatory response

Doses of NMs were first expressed in μg/cm^2^, after normalization of the deposited doses by the total alveolar surface in vivo (4000 cm^2^) or by the surface of the cell layer in vitro. All the LOAELs and the dose intervals determined for pro-inflammatory effects were expressed using this dose metric (Tables 3 and 4, Fig. 4a) and *vivo-vitro* comparisons were performed. Generally, for each NM, we noted pro-inflammatory effects at lower doses in vivo compared to in vitro. We also observed that the LOAELs and the dose intervals determined in vitro after exposure at the ALI were closer to those in vivo than those determined in vitro in submerged conditions. Moreover, we noted that the LOAELs determined in vitro were closer to those in vivo when the final dose was achieved in vitro within 3 h rather than within 24 h. When comparing the LOAELs for each NM, differences of a factor of 10, 30 and 100 were noted for exposure at the ALI to aerosols (3 h + 21 h), exposure in submerged conditions in inserts (3 h + 21 h) and in submerged conditions in plates (24 h), compared to in vivo, respectively.

**Table 4:**
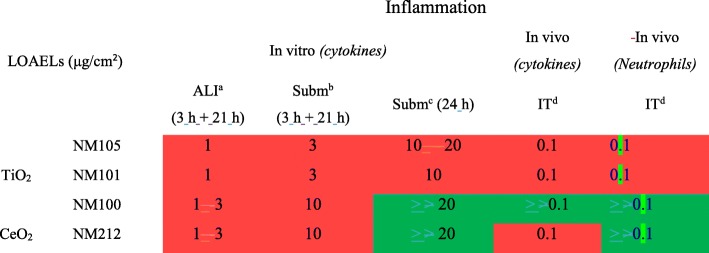
LOAELs (in μg/cm^2^ for 24 h of exposure) determined for pro-inflammatory effects

Significant effects allowing the determination of a LOAEL

No significant adverse effects observed

^a^Doses tested at the ALI: 0.1, 1, 3 μg/cm^2^

^b^Doses tested in submerged conditions in inserts: 1, 3, 10 μg/cm^2^

^c^Doses tested in submerged conditions in plates: 1, 3, 10, 20 μg/cm^2^

^d^Doses tested in vivo: 0.001, 0.01, 0.1 μg/cm^2^

#### Comparisons in mass/macrophages

Doses were also normalized by the total number of macrophages and expressed in μg/10^6^ macrophages to compare in vivo and in vitro LOAELs and dose intervals (Table 5, Fig. 4b and Additional file 1: Table S3). For that purpose, in vivo doses expressed in μg were normalized by the number of alveolar macrophages. In vitro, deposited doses expressed in μg/cm^2^ were normalized by the total number of alveolar macrophages-like cells per cm^2^. We noticed that the LOAELs and the dose intervals determined in vitro were closer to those observed in vivo when the doses were normalized by the number of macrophages rather than by the alveolar surface (Table 5 and Fig. 4). When looking at the LOAELs, the pro-inflammatory responses were observed at similar doses in vivo and in vitro at the ALI, whereas a difference of at least a factor of 10 was observed when the LOAELs were expressed in μg/cm^2^. Differences of around a factor of 3 and 20 were observed between the in vivo experiments and the in vitro experiments performed in submerged conditions in inserts (3 h + 21 h) and in plates (24 h), respectively.

**Table 5:**
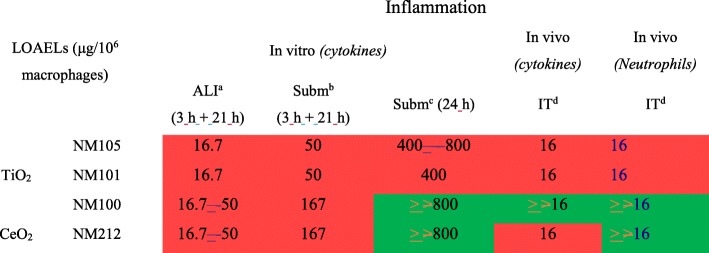
LOAELs (μg/10^6^ macrophages for 24 h exposure) determined for pro-inflammatory-effects

Significant effects allowing the determination of a LOAEL

No significant adverse effects observed

^a^Doses tested at the ALI: 1.67, 16.7, 50 μg/10^6^ macrophages

^b^Doses tested in submerged conditions in inserts: 16.7, 50, 167 μg/10^6^ macrophages

^c^Doses tested in submerged conditions in plates: 40, 120, 400, 800 μg/10^6^ macrophages

^d^Doses tested in vivo: 0.16, 1.6, 16 μg/10^6^ macrophages

### Ranking of the NMs according to the methodology used

**Fig. 5:**
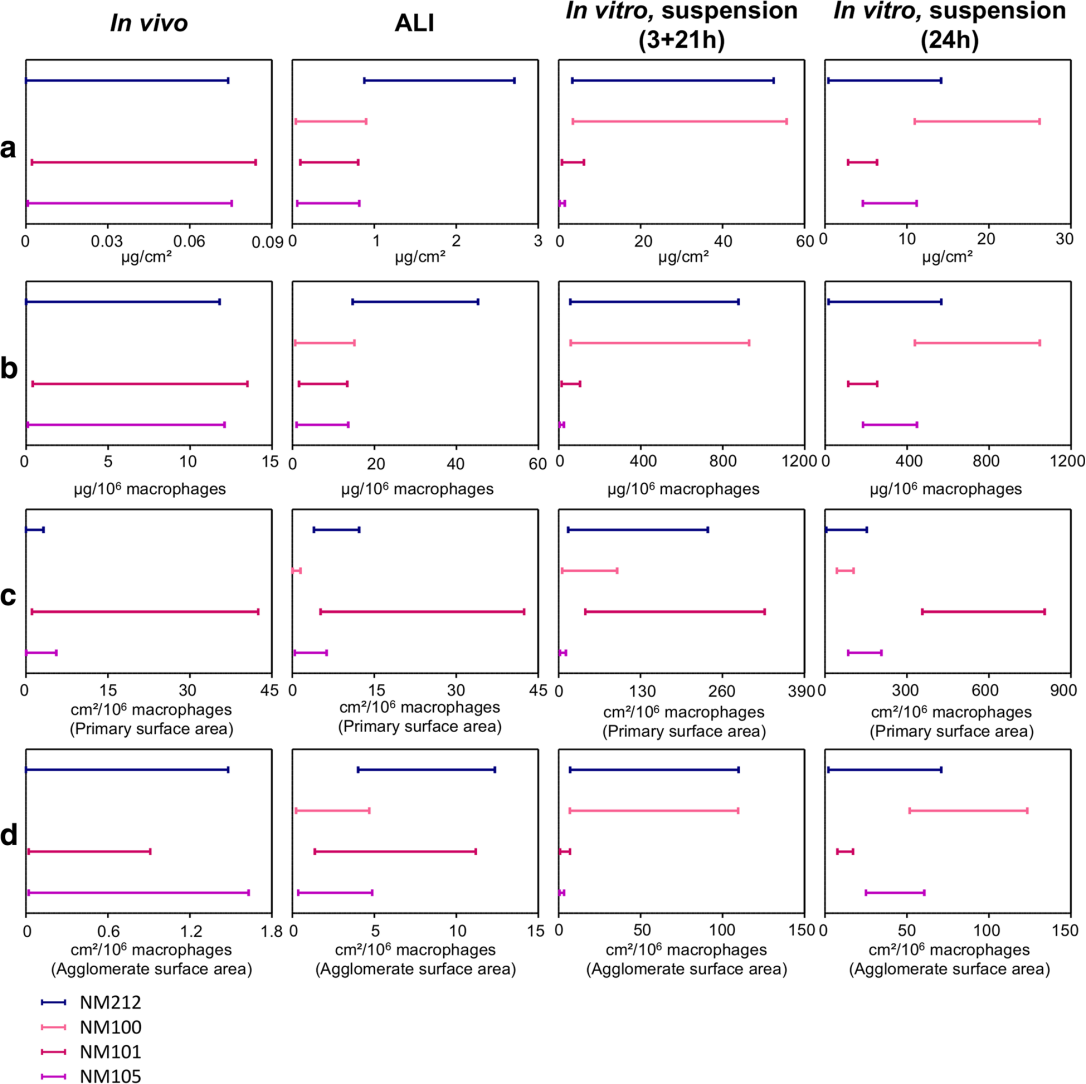
Dose intervals of NMs for inflammation according to methodologies and dose metrics used. Dose intervals calculated for general acute pro-inflammatory response were used to compare the ranking of each NM in function of each exposure method used. Comparisons were also performed according to the four dose metrics used in our study (**a**: mass/alveolar surface), (**b**: mass/macrophages), (**c**: dose in mass/macrophages normalized by primary surface area), (**d**: dose in mass/macrophages normalized by agglomerate surface area). In vitro, alveolar epithelial cells in co-culture with macrophages were exposed for 24 h at the air-liquid interface (ALI) or in submerged conditions to suspensions of NMs. In vivo, rats were exposed by intratracheal instillation of NM suspensions. After 24 h of exposure, the biological activity was assessed, focusing on pro-inflammatory mediators. For each exposure method, each NM and each cytokine, benchmark dose-response modeling was used to estimate the critical dose related to a 20% increase of pro-inflammatory mediator level and the lowest (BMDL) and the highest (BMDU) dose of the interval corresponding to a confidence interval of 90%. A median dose intervals was then calculated by pooling the dose intervals of the four cytokine to have a general pro-inflammatory response

For each methodology used, a ranking of the four NMs used was provided according to the inflammation results and the dose intervals that had been determined. Ranking comparisons were performed using dose intervals only, because better screening could be performed between NMs by using this criterion of effect compared to the use of LOAELs. Comparisons were performed to assess whether the four poorly toxic and poorly soluble NMs could be ranked similarly, based on the different methodologies tested. The dose intervals were also normalized by NM primary surface areas and agglomerate surface areas to understand the differences in toxicity existing between the NMs. A toxicity ranking of the NMs according to the different methodologies and dose metrics used is presented in the Fig. 5.

#### Ranking using mass as dose metric

In mass (μg/cm^2^ or μg/10^6^ macrophages) some differences were observed between in vivo and in vitro conditions (Fig. 5). In vivo, NMs 105, 101 and 212 were observed to be clearly more toxic than NM100, as we did not observe any significant effects with the NM100. In vitro, we noticed pro-inflammatory responses for NMs 105 and 101, at lower doses than for NM212, at the ALI and in submerged conditions. NM100, similarly as NM105 and NM101, seemed to elicit more pro-inflammatory responses at the ALI than NM212, but this was not observed in submerged conditions.

#### Ranking using the surface area as dose metric

Doses in mass/macrophages were normalized by the surface area of each NM, to assess how the surface reactivity influenced the biological responses in vivo and in vitro. The dose intervals were expressed in cm^2^/10^6^ macrophages (Additional file 1: Tables S4 and S5) and a ranking was provided for the four poorly toxic and poorly soluble NMs used in our study. Doses in mass/alveolar surface could also be normalized by the surface area, generating the same ranking of the NMs as using the mass/macrophages dose metric (Fig. 5).

First, the dose intervals were normalized by the calculated surface area using the NM primary sizes and densities (BET method) (Additional file 1: Table S4). This normalization had an influence on the ranking of the NMs. The NM101 was ranked with a lower toxicity than expected, both in vivo and in vitro (Fig. 5). In vivo, we observed dose intervals at lower doses for NM105 and NM212 than for NM101, however, it was not possible to include NM100 in this ranking, as no significant effects were observed, probably because significantly lower doses (in cm^2^/10^6^ macrophages) were tested compared to the three other NMs, due to a lower surface area. In vitro, when dose intervals were expressed in cm^2^/10^6^ macrophages, using surface area calculated according to primary sizes, we also observed effects at lower doses for the NM100, the NM105 and the NM212 than for the NM101.

Secondly, the dose intervals were normalized by the surface area calculated according to NM agglomerate mean sizes and densities in exposure media (suspensions or aerosols) (Additional file 1: Table S5). Interestingly, less changes in the ranking of the NMs were observed when performing this normalization, for all the methodologies used (in vitro ALI, in vitro in submerged and in vivo) (Fig. 5). In vivo, no clear discrepancy could be made between NMs 105, 101 and 212; all three were observed to be more toxic than NM100. In vitro at the ALI, NMs 105, 101 and 100 were observed toxic at lower dose than NM 212, although this was clearly more pronounced for NM105 and NM100. In submerged conditions, similarly as when the dose intervals were expressed in mass/macrophages, NMs 105 and 101 were observed to be more toxic than NMs 100 and 212. This better correlation in the ranking between doses expressed in mass and doses expressed in surface area, when normalizing the dose intervals by mean agglomerates surface area rather than by primary surface areas, indicates that toxicity may be due to NM agglomerates rather than isolated NMs.

## Discussion

The aim of this study was to assess the ability of several more or less advanced in vitro methods, to predict the pulmonary adverse effects observed in vivo after acute exposure to poorly toxic and poorly soluble metallic NMs. The perspective is to promote reliable alternative methodologies to animal testing for the prediction of pulmonary toxicity of NMs in humans.

The selection of relevant in vivo and in vitro models to predict potential biological responses in humans was thus very important. In vivo, the rat was selected because it is the recommended species to assess inhalation toxicity in humans [21]. In vitro, human rather than rat cells were chosen because they were more likely to model responses of cells in the human body. Moreover, because the principal pulmonary target for inhaled NMs remains the alveoli [22], we focused on this part of the lungs. At the alveolar surface in vivo, macrophages are in close contact with epithelial cells (i.e type and I and II pneumocytes), at a ratio of approximately one macrophage to ten pneumocytes [18, 20]. The main role of the macrophage is to engulf particles to eliminate them from the alveolar space [23]. Type I pneumocytes serve as a thin gas-permeable epithelial barrier [24]. Type II pneumocytes have a role in defense of the alveoli thanks to their physiological abilities [25]. In the alveoli, macrophages and epithelial cells are at the ALI and are covered by a thin layer of surfactant secreted at the apical side by the type II pneumocytes.

To mimic the cell organization at the alveolar surface and the potential interactions between the cells and the NMs, a co-culture using two cell types was selected in vitro. The A549 alveolar epithelial cell line was selected for its ability to form a cell layer and to secrete surfactant [23, 26]. The THP-1 monocyte cell line was chosen for its capacity to differentiate into macrophage-like cells with Phorbol Myristate Acetate (PMA) [27]. This model was selected for its increased sensitivity compared to mono-cultures of alveolar epithelial cells at the ALI [13] and in submerged conditions [28–30]. We also postulated that with this co-culture model, the deposited NMs could become covered with surfactant before they interact with the cells, as observed in the alveoli in vivo [22]. Nevertheless, because the presence of surfactant was not evaluated in our study, it remains unclear whether the cells and the NMs were covered by surfactant.

Different exposure methods were assessed. In vivo, rats were exposed for 24 h by intratracheal instillation of NM suspensions, after hyperventilation. In vitro, cells were exposed using more or less advanced methods. Co-cultures were exposed at the ALI in inserts to simulate more closely the interactions between NMs and alveolar cells occurring in vivo, and to avoid contamination with culture medium. The ALI exposure system used in our study [13] was selected for its ability to deposit sufficient amounts of NMs on cells to observe biological adverse effects [31, 32]. In parallel we also used a more classical exposure method and cells were exposed to NM suspensions in submerged conditions to assess the general impact of the culture medium surrounding poorly soluble NMs on the cell biological response.

Both in vivo and in vitro, the toxicity was assessed after 24 h of exposure to the same TiO_2_ and CeO_2_ NMs. For that, we focused on the deposited doses on cells or into the lungs, because metallic and poorly soluble NMs exert their toxicity mainly by direct contact with the cells [17]. Moreover, we tested different timings of the dose delivery in vitro, to assess if that factor could influence the cell response. In vivo, the final doses of NMs were deposited by instillation almost instantly into the lungs. At the ALI in vitro, NMs were deposited on the cells using a very low aerosol flow rate of 5 mL/min to prevent cell damages due to the air flux. To deposit a dose sufficient to observe biological effects, cells were exposed for 3 h to aerosols. After exposure, the cells were then kept in the incubator for the remaining 24 h with NMs deposited on their surface. In submerged conditions, it was not possible to deposit the NMs instantly on the cells either, as the deposition kinetics depended mostly on their sedimentation rate. In inserts, we used the duration of 3 h for the dose delivery, in order to provide comparisons as accurate as possible between ALI and submerged exposure. As for the ALI, the cells were kept for the remaining 21 h with NMs deposited on their surface. In submerged conditions in plates, we used classical exposure conditions and the NM deposition on the cells was maintained for 24 h.

Similar endpoints were selected to compare in vivo and in vitro toxicity. The biological responses were assessed after 24 h of exposure to the NMs, by performing cytotoxicity, oxidative stress and inflammation assays, to determine the absolute toxicity of each NM. Both in vivo and in vitro, we observed that inflammation was the most sensitive parameter for detection of biological responses at 24 h. After NM exposure, we detected significantly more pro-inflammatory effects than cytotoxicity and oxidative stress responses, and generally at lower doses. We were not surprised about the absence of clear pulmonary cytotoxic effects in our study as poorly soluble TiO_2_ and CeO_2_ NMs were shown to be not very cytotoxic at 24 h both in vivo and in vitro [33]. Regarding oxidative stress production, as ROS are known to interact quickly with molecules present in the cells, better detection could have been achieved by performing several measurements during the 24 h of exposure. Moreover, in our protocol, cells were incubated with DCFDA probe after exposure and not before exposure which may have reduced assay sensitivity [34]. Nevertheless, in absence of cytotoxicity at 24 h, which is the case in our study, the authors did not show a clear increase of ROS measurement sensitivity when the probe was added before NM exposure [34].

For these reasons, comparisons were performed mostly with inflammation markers as readout for NM toxicity. Nevertheless, it has to be noted that the inflammatory effects were observed at high doses (at least 10-fold higher) compared to realistic human exposure scenarios [7]. The markers of inflammation used in our study were selected according to their relevance in representing the pro-inflammatory acute response in the lungs after exposure to particles [35, 36]. Similar pro-inflammatory mediators were chosen in vitro and in vivo, however those significantly secreted in vitro were not necessarily predominant in vivo. On the basis of this finding we assumed that better comparisons could be provided in our study by considering the global inflammatory response, more particularly the secretion of pro-inflammatory mediators that can be measured both in vivo and in vitro.

To perform quantitative comparisons, LOAELs were first determined according to the significant pro-inflammatory responses observed. Secondly, we used benchmark dose-response modeling to determine dose intervals related to increase in pro-inflammatory mediator levels. There are multiple advantages of using benchmark dose modeling instead of a No Observed Adverse Level (NOAEL) or LOAEL approach in hazard assessment. Using NOAEL/LOAEL approach, toxic effects are reduced to a yes/no question and are typically determined based on the presence or absence of statistical significance. Moreover, the uncertainty in the NOAELs or LOAELs cannot be quantified and can be considerable as it sticks only to the dose tested. Thus NOAELs and LOAELS determined depend strongly on the study design [37–39]. On the contrary, dose intervals determined by benchmark modeling take the potency of NMs to induce an effect into account and take uncertainty in the data into account. The question is not whether an effect is induced or not, but at what dose an effect of interest is induced. These dose intervals provide more accurate comparisons between in vivo and in vitro data. [37–39].

Although there were clear advantages in using dose intervals, some uncertainties remain in our study regarding their determination due to limited log dose data intervals and because the pro-inflammatory effects were observed mostly at the highest doses tested. This highlights the importance of providing experimental data that should allow to model a reasonable response slope by either providing a clear dose-response pattern of toxicity (that implies the use of toxic compounds) or more refined tested doses in case of low toxicity. The more experimental doses are tested, the more accurate is the analysis and this is true also for NOAEL/LOAEL assessment.

Comparisons were performed with LOAELs and dose intervals to assess if similar conclusions could be made using the two criteria of effect dose. As the LOAELs and dose intervals were associated with dose metrics, the key point for the comparisons was to select similar metrics for both in vivo and in vitro methodologies. For that, we focused on the deposited masses because it take into account the direct contact between the NMs and the tissues, that was shown to be the main cause of toxicity for poorly soluble NMs [15–17]. Doses in mass were first normalized to the total alveolar surface in vivo or to the surface of the cell layer in vitro. This normalization was based on the assumption that the alveolar epithelium may be the main target after acute exposure to NMs [14, 22].

Expressing results in mass/alveolar surface, we observed responses at doses around ten times lower in vivo (LOAELs at 0.1 μg/cm^2^ and BMDU around 0.08 μg/cm^2^) than in vitro at the ALI (LOAELs at 1 μg/cm^2^, BMDU from 0.8 to 2.7 μg/cm^2^). The differences were slightly more pronounced in submerged conditions when the dose was delivered in 3 h (at least 20-fold compared to in vivo) (LOAELs at 3 μg/cm^2^, BMDU from 1.5 to 55 μg/cm^2^), and much more important, with a factor of around 100, when the deposition of the NMs was continuous during the 24 h of in vitro exposure (LOAELs at 10 μg/cm^2^, BMDU from 6.4 to 26 μg/cm^2^). Interestingly, when comparing in vivo and in vitro biological activation levels using the mass/alveolar surface metric, similar differences were observed with LOAELs and dose intervals determined using benchmark dose-response modeling. This indicates that similar conclusions could be made using these two criteria of effect, when comparing biological activation levels in vivo and in vitro.

In vivo and in vitro results expressed in mass/alveolar surface were also compared in three noteworthy studies [10, 11, 40]. In their study, Kim et al. [10] observed similar pro-inflammatory profiles when expressing the doses in μg/cm^2^, after exposing mice by oropharyngeal aspiration and macrophages or lung slices to suspensions of TiO_2_, CeO_2_ and SiO_2_ NMs. Nevertheless, the real masses of NMs deposited in vitro and ex vivo were not assessed and no clear quantitative comparisons were performed between the in vivo and the in vitro approaches, which renders the interpretation of the results from their study difficult. Jing et al. [40] compared the responses after acute exposure to Cu NPs, in mice lungs and in alveolar epithelial cells at the ALI. They observed similar responses (chemokine and LDH release) but apparently at lower doses in vitro compared to in vivo, when expressing the dose in ng/cm^2^. Nevertheless, the responses were assessed at 2 h or 4 h in vitro and at 24 h or 40 h in vivo, which brings uncertainties towards their comparative results. Teeguarden et al. [11] exposed mice in vivo by inhalation or lung epithelial cells in vitro with suspensions of FeO NMs for 4 h with similar timings of the dose delivery and assessed the mass and regional deposition of the NMs. When focusing on the tracheobronchal part of the lung, they observed pro-inflammatory responses with doses about 10 to 100-time lower in vivo than in vitro. However, they did not study the response of the alveolar part of the lung with this metric. Instead, they normalized the doses in mass by the number of alveolar macrophages in vivo and in vitro. Indeed, monocultures of murine macrophages were also exposed to suspensions in their study [11]. When focusing on the alveolar macrophages, they showed that pro-inflammatory responses were triggered at similar doses in vitro and in vivo.

Interestingly, we also observed significant effects at much closer doses in vivo (LOAELs at 16 μg/10^6^ macrophages, BMDU around 12 μg/10^6^ macrophages) and in vitro using this dose metric, and more particularly at the ALI (LOAELs at 16.7 μg/10^6^ macrophages, BMDU from 13 to 45 μg/10^6^ macrophages) and when the dose was deposited in submerged conditions on the cells within 3 h (LOAELs at 50 μg/10^6^ macrophages, BMDU from 25 to 900 μg/10^6^ macrophage), rather than in 24 h (LOAELs at 400 μg/10^6^ macrophages, BMDU from 250 to 1000 μg/10^6^ macrophage). As shown by using the mass/alveolar surface metric, similar differences were observed using LOAELs and dose intervals, when comparing in vivo and in vitro biological activation levels using the mass/macrophage metric. Nevertheless, for the NMs 100 and 212 the differences of toxicity observed between submerged exposure in inserts for 3 h and submerged exposure in plates for 24 h were less obvious when looking at the interval of doses instead of the LOAELs. Finally, pro-inflammatory effects were still observed at higher doses in vitro in submerged conditions compared to at the ALI or in vivo. Although serum was added in our in vitro experiments in submerged conditions to keep the cells in their best physiological conditions, we hypothesized that the presence of serum may have reduced potential NM toxicity. Indeed, it has been shown that NMs were less toxic in vitro in presence of serum in suspensions compared to in absence of serum [41, 42]. Taking that into account, better correlations at 24 h may be provided in absence of serum, however this point still has to be demonstrated.

Although differences exist regarding the cellular and animal models and the duration of exposure between the Teeguarden study and ours, this seems to indicate that focusing on the cell number might better explain the general acute pro-inflammatory response elicited by NMs in the alveoli, both in vivo and in vitro. However, in our study we did not discriminate between the responses from the alveolar epithelial cells and the macrophages. Moreover, it should be noted that although a ratio of one macrophage for ten pneumocytes was used to mimic in vitro the ratio present in vivo in rat or human lungs [18], the number of macrophages/cm^2^ (60,000/cm^2^ in inserts or 25,000/cm^2^ in plates) was higher in vitro compared to in vivo (around 6000 macrophages/cm^2^) because the A549 cell surface in vitro was much lower than the alveolar epithelial cell surface in vivo. This could explain why in vivo and in vitro results were matching better when the doses were expressed in mass/macrophages rather by in mass/alveolar surface.

Ranking comparisons were also performed between the four NMs tested. For ranking comparisons, we focused on dose intervals only. Indeed, LOAELs are depending a lot on the experimental design as they are strictly determined according to the doses tested. In the case of few doses are tested and when NMs are observed to be toxic only at the highest doses tested, like in our study, LOAELs do not allow to make clear differences between the NMs. However, with dose intervals determined using benchmark dose-response modeling, more accurate effect doses were determined for each NM and each exposure method. Thanks to this criterion of dose, a better screening of the four NMs has been performed. The comparisons were performed using the mass metric but also with the surface area metric, since it was shown that the surface area was the most effective dose metric to explain acute NM toxicity in the lung [16, 17, 43].

Expressing the doses in mass (mass/alveolar surface or mass/macrophages), similarity in the rankings were observed between in vivo and in vitro conditions for the three TiO_2_ NMs. Both in vivo and in vitro, NMs 105 and 101 appeared more toxic than NM100, except at the ALI were similar toxicity was observed for the three TiO_2_ NMs. However, it was not the case for the CeO_2_ NM212. NM212 was observed as toxic as NMs 105 and 101 and more toxic than NM100 in vivo, whereas it was observed to be less toxic than the TiO_2_ NMs 105 and 101 in vitro. Moreover, we noticed that the ranking of the NMs could change according to the dose metric used. Generally, when using the NM mass as dose metric, the NM101 appeared as toxic as the NM105 and more toxic than the NMs 212 and 100. Nevertheless, although we observed similarities in nanomaterial rankings between in vivo and in vitro approaches, benchmark dose intervals were too large to make clear ranking comparisons, due to the insufficient quality of the data-set. This underlines the importance of providing good quality data to perform reliable comparisons. Because of the insufficient quality of the data-set, it remains thus undetermined if ALI exposure methods could provide better predictivity than submerged methods regarding the ranking of the NMs.

When doses in mass were normalized by NM primary surface areas, the NM101, that has the highest surface activity, was observed to be less toxic than expected and clearly appeared less toxic than the other NMs. Indeed, based on the surface reactivity theory which implies that higher NM surface areas induce higher potential toxicities [44], similar responses were to be expected from these three NMs when normalizing the dose by surface area. This has been shown in vivo [45] and in vitro [16]. Because this was not the case, we hypothesized that the hydrophobic surface coating that surrounds the NM101 but not NM105, NM100 and NM212 may have contributed to reduce the toxic potential of NM101. This was not surprising as it was shown in several studies that NM acute toxicity was more dependent on coating than on core properties [46, 47].

However, when the doses in mass/macrophages were normalized by surface areas calculated using mean agglomerate sizes and densities, we did not observe this clear change in the NM101 ranking. Indeed, similarities in the rankings were observed between doses expressed in mass/macrophages and in cm^2^/macrophages when surface areas were calculated using mean agglomerate sizes and densities. This indicates that focusing on mean surface areas in exposure media rather than on primary surface areas may better explain the biological responses observed with poorly soluble NMs. Nevertheless, further investigations are necessary to confirm this allegation.

Comparing several in vitro methods to the in vivo approach, that was considered as the reference method in our study to estimate the potential toxicity of NMs in humans, allowed us to evaluate the predictive ability of different in vitro system in absolute terms. Finally, according to our results, it seems that the use of advanced and realistic in vitro methodologies allows to predict more closely the biological responses observed in vivo and thus might give a better estimation of the potential absolute toxicity in humans.

Nevertheless, further improvements still need to be made to draw clear conclusions. In our study, the animals were exposed by suspension instillation and not by inhalation of aerosols containing NMs. The instillation method remains less physiologic than the inhalation route, especially because the dose is instantly deposited into the lungs using a bolus. This could induce a greater biological response compared to inhalation, where the final dose is generally deposited within 4 h [48]. Moreover, although the instillation method allows to deposit NMs more deeply into the lungs [49], there was a lack of accurate dosimetry in our study as the Multiple-Path Particle Dosimetry Model (MPPD) [50] could not be used. Thus, the regional deposition and more particularly the real dose distributed to the alveoli was not accurately evaluated.

Furthermore, some limitations remain regarding the assessment of dose delivery in vitro, more particularly in submerged conditions as the deposited dose on cells was estimated using the ISDD model and not directly measured. Nevertheless, the relative uncertainty was probably low as good similarities were observed between the estimated and measured deposited doses of poorly soluble NMs at 24 h [51]. At 3 h, the uncertainty may have been higher and could have led to an underestimation of the deposited dose [51]. This may have contributed to increased differences between the ALI and the submerged exposures in terms of biological activation levels.

Another reason why it is difficult to conclude clearly that the use of advanced and realistic in vitro methodologies might give a better estimation of the potential absolute toxicity in humans is that some uncertainties exist regarding the dose metrics selected. To compare the in vivo to the in vitro approach, we normalized the dose in vivo in mass by the total alveolar surface. We decided to use the value of 4000 cm^2^ [18, 19], which seemed suitable for 7 weeks old male rats. Nevertheless, this may represent an overestimation as alveolar surfaces of around 2000 [52], and 3400 cm^2^ [53] have also been calculated for 6 weeks and 60 days old rats, respectively. To normalize the dose by the number of macrophages, we assumed that around 25 million of macrophages were in the alveoli in vivo and we used the number of counted macrophages in vitro. Although we based ourselves on two publications [18, 54] to determine the number of alveolar macrophages in vivo, it remains unclear whether all of them were in contact and contributed to the biological response elicited by the NMs, more particularly considering that only around 8 million of macrophages were retrieved in the BALF in vivo. Nevertheless, we decided to use the value of 25 million of macrophages instead of the measured value of 8 million because we observed that the number of macrophages retrieved from the BALF was depending a lot on the experimenter and because the protocol used in our study was not implemented to retrieve all the alveolar macrophages.

Some uncertainties also remain because our experimental data-set did not allow to provide a clear dose-response pattern of toxicity. That may had an impact on the accuracy of our comparisons. For example in vivo, there was a difference of a factor of ten between each dose tested; this might prevent us to determine accurate LOAELs and dose intervals. This is particularly true because the pro-inflammatory effects were observed at the highest dose tested. Although using intermediary doses might have enabled to determine more precisely LOAELs and critical dose intervals, this has no impact on our general conclusions regarding comparison of biological activation levels between the different exposure methods used in our study: regardless the criterion of comparison used, the in vivo methodology remains the most sensitive one in our study, to predict potential adverse effects after acute exposure to poorly soluble NMs. Regarding NM rankings, we observed that it was difficult to use LOAELs to rank NMs in function of each exposure methodology used and that determining dose intervals using benchmark dose-response modeling was very important for this purpose. However, because the data-set quality used in our study was not optimal, the dose intervals determined were too large to provide clear and reliable comparisons of NM rankings between each methodology used. To perform in vivo *-* in vitro comparisons we thus recommend to test more doses and to reduce the interval between each doses, in order to determine more accurate dose intervals.

## Conclusion

Quantitative comparisons were performed between in vivo and in vitro acute pro-inflammatory responses using compatible dose metrics. Biological activation levels were compared and we showed better in vivo- in vitro correlations when doses were expressed in mass/macrophages rather than in mass/alveolar surface. Using the determined LOAELs and critical effect dose intervals, we assessed the ability of each in vitro method used in our study to predict the biological responses in vivo. We showed that the most realistic in vitro exposure method: the ALI method, was the most predictive in terms of absolute toxicity, whatever the dose metric used. In vitro, we also showed better *vivo-vitro* correlations while using timings of dose delivery of 3 h rather than 24 h. For each exposure method, we ranked NMs in function of their toxicity and we highlighted that critical effect dose intervals could be used instead of LOAELs to provide more accurate comparisons between the NMs. Regarding toxicity rankings of NMs, relative similarities were shown between in vivo and in vitro methodologies. Nevertheless, we could not conclude clearly about each in vitro methodology ability to predict the NM rankings observed in vivo because the quality of the data-set was insufficient to determine accurate dose intervals. Interestingly, we also observed when normalizing the doses by NM surface areas, that the toxic effects were probably more attributable to agglomerates, rather than to isolate NMs.

In conclusion, we showed that advanced methods could be used to enhance the in vitro experiments ability to predict potential acute pulmonary toxicity in vivo. Moreover, we highlighted that careful consideration of some key methodological points in vitro could contribute to improve in vitro methods predictivity, including control of the timing of the dose delivery. Although these conclusions are inferred from our experimental data-set and should be further confirmed with other nanomaterials, including more toxic NMs, this study brings new perspectives regarding the usage and development of advanced in vitro methods.

## Methods

### Nanomaterials

Four poorly toxic and poorly soluble NMs were used in the study. The TiO_2_ NM100 and NM101 and the CeO_2_ NM212 were obtained from the Joint research center (JRC). The TiO_2_ NM105 was obtained from Evonik Industries (AEROXIDE® TiO_2_ P25). Data indicated in our study regarding primary sizes and specific surface areas (BET) were provided by the manufacturer (Table 1). TiO_2_ and CeO_2_ primary physico-chemical properties were also well characterized by the JRC. [55, 56]. The endotoxin levels of the NMs were tested by partners of the European project NANoREG. They were below the limit of detection (data not shown).

### In vivo study

#### Animals

Pathogens free 7 weeks old male rats (WISTAR RjHan:WI, JANVIER LABS, France; 250 g), were housed in polycarbonate cages, in a temperature and humidity controlled room, and had free access to food and water ad libitum. All the in vivo experiments were approved by the “Comité Régional d’Ethique en Matière d’Expérimentation Animale de Picardie” (CREMEAP) (C2EA – 96).

#### Preparation of NM suspensions

Similarly as for the in vitro study, suspensions of TiO_2_ (NM105, NM101, NM100) and CeO_2_ (NM212) at 10 mg/mL in Mili-Q water were prepared and then sonicated at amplitude 100 during 2 min (1 min on, 1 min off, 1 min on) using a cuphorn sonicator (QSONICA, Q700). Suspensions in Milli-Q water at 5; 0.5 and 0.05 mg/mL were prepared to expose rats to 500; 50 and 5 μg/animal, respectively.

#### Characterization of NM suspensions

For each NM, DLS measurements were performed (Malvern, Zetasizer Nano S) on NM suspensions to measure the hydrodynamic diameter and to assess the size distribution of the particles in suspensions. DLS results on water suspensions used to instill animals are presented in the Additional files section (Additional file 1: Figure S4). Regarding in vitro experiments, DLS measurements were performed after sonication in stock suspensions (2.56 mg/mL in milli-Q water) and just after dilution in 0.4 mg/mL suspensions in culture medium. These in vitro results were presented in our previous article [13]. Surprisingly, similar results were observed between NM suspended in water and in culture medium.

#### Intratracheal instillation

Rats were anesthetized (0.5 mg/kg ketamine hydrochloride, 0.1 mg/kg atropine and 1 mg/kg xylazine), endotracheal intubation was performed using a canula and animals were connected to a respirator (Harvard Apparatus, ventilator model 683) for 30 s to create a hyperventilation. Rats were disconnected from the apparatus, 100 μL of NMs suspension in water or vehicle (Mili-Q water) was added in the cannula and suspensions were directly aerosolized into rat lungs by physiological aspiration. It was chosen to disperse NMs in Milli-Q water and not in physiological saline buffer to enhance NM stability in suspension. This choice was made since it was shown that intratracheal instillation of distilled water in rats, like physiological saline, did not induce significant inflammatory responses at 24 h [9].

#### Dosing and biodistribution analysis

After instillation, rats were sacrificed 3 h after instillation to evaluate the lung burden (*n* = 2). Mass of NM was measured in collected lungs by inductively coupled plasma mass spectrometry (ICP-MS) analysis. Briefly, a procedure consisting of incubation with a mixture of nitric acid (HNO3) and hydrofluoric acid (HF), and heating was applied to digest lungs and TiO_2_ nanomaterials in order to determine the total Ti content by ICP-MS [57].

#### Assessment of biological activity

Animals (*n* = 6) were sacrificed 24 h after instillation and bronchoalveolar lavages were performed with PBS. A first bronchoalveolar lavage was performed using 5 mL of PBS for biochemical analysis. Two other lavages were then performed with 10 mL of PBS to collect more cells. Collected BALFs were centrifuged at 350 g for 10 min and 4 °C, to separate the cells from the supernatant. The supernatants recovered from the first lavage (around 4.5 mL for each sample) were aliquoted in eppendorf tubes and stored at − 80 °C until analysis.

##### Cell counting

After centrifugation, the cell pellets were resuspended in 5 mL of RPMI medium (Gibco, 61870), 20 μL of cell suspension were mixed with 20 μL of propidium iodide containing accridine solution (Nexcelom, CS2-0106) and the cells were counted using a cell counter equipped with a fluorometer (Nexcelom, Cellometer® Auto 2000), to differentiate the dead cells and the erythrocytes from the pulmonary cells.

##### BALF cytology

After counting, the cells were diluted in RPMI, seeded on slides at 300000 cells/spots using a cytospin (300 g, 5 min) (Shandon, cytospin2) and then fixated and coloured in May-Grunwald Giemsa (MGG). Briefly, the slides were fixated in MG pure for 3 min followed by 2 min in MG diluted at 50% in Mili-Q water, rinsed 2 times with Mili-Q water for 20 min and then coloured in Giemsa. The percentage of the different cell types (macrophages, neutrophils, eosinophil) in BALF was then determined using optical microscopy.

##### Intracellular ROS levels (DCF assay)

After counting, the BALF cells were seeded at 1 × 10^6^ cells/mL in 24 well plates (Falcon, 353047) (in RPMI medium supplemented with 10% of FCS: 0,5 mL/well), and were then incubated for 18 h at 37 °C and with 5% of CO_2_ to let the cells (mostly macrophages) to adhere on the plate. The cells were then rinsed with PBS and incubated for 35 min with 10 μM of 5-(and-6)-chloromethyl-2′,7′-dichlorodihydrofluorescein diacetate (CM-H_2_DCFDA) probe (Life technologies, C6827) in PBS (0.5 mL/well). After 30 min of incubation, the probe was removed in some control wells, 1 mM of H_2_O_2_ in PBS was added and the cells were incubated for 5 min, to serve as positive control. After incubation, the cells were washed with PBS and incubated for 5 min in 90% of Dimethyl Sulfoxide (DMSO) (Sigma-Aldrich, D2438) in PBS (0.5 mL/well). The cells were then scraped using scrapers (TPP, 99002), the well contents were retrieved in tubes (Eppendorf, 3810X) and the tubes were centrifuged at 10000 g and 4 °C for 5 min, to eliminate the dead cells and to remove the remnants particles. The tube contents were transferred in 96 well black plate (150 μL/well) (Greiner Bio-one, 655076) and the fluorescence of the samples was read (excitation: 488 nm, emission: 530 nm) using a spectrophotometer (TECAN, infinite 2000). The value of each sample was expressed in percentage of intracellular ROS compared to the control.

##### Pro-inflammatory release in BALF supernatants

Il-1β, IL-6, IL-8 and TNF-α releases were measured in collected supernatants using a commercial available ELISA multiplex kit (Mesoscale discovery, Proinflammatory Panel 2, N05059A-1) and a multiplex reader (Mesoscale discovery, Sector Imager 24000) according to supplier recommendations.

##### LDH release and protein levels in BALF supernatants

LDH release were quantified in BALF using a commercially available kit (Promega, CytoTox-ONE Homogeneous Membrane Integrity assay). Proteins levels were measured in BALF using a Bradford assay (Biorad, protein assay kit).

##### Statistical analysis

All data were expressed as mean ± standard deviation (SD) (*n* = 6). Statistical analyses were performed using Graphpad Prism 5.0 (GraphPad Software Inc., San Diego, CA). Results were analyzed by a non-parametric Kruskal-Wallis test followed by Dunn’s post-hoc test to compare the different treated groups to the non-exposed control.

### In vitro study

All materials and methods used in the in vitro study are fully detailed in the following article [13]. Briefly, alveolar epithelial cells (A549) in co-culture with macrophages (THP-1) were exposed either at the ALI to aerosols or in submerged condition to suspensions of TiO_2_ and CeO_2_ NMs. A ratio of ten A549 for one THP-1 was used to mimic the ratio existing in vivo in the lungs.

Different timings of the dose delivery were used in vitro. At the ALI, cells were exposed to aerosol of NMs using a Vitrocell® system. In this system, the NM deposition was maintained for 3 h, meaning that the final dose was reached within 3 h. The cells were then kept in the incubator for the remaining 21 h at the ALI with the NMs deposited on their surface. In submerged conditions, two different dose rates were used. Cells were exposed to suspensions of NMs in inserts using similar timing of the dose delivery as at the ALI. The NM deposition was maintained for 3 h. After 3 h of exposure, the deposition was stopped by replacing NM suspensions by fresh medium and cells were then kept during the remaining 21 h in submerged condition in the incubator. Cells were also exposed in plates to suspensions of NMs for 24 h, to represent the exposure conditions usually used in vitro. In that situation, the NM deposition was maintained for the whole exposure time, meaning that the final dose was reached within 24 h. After 24 h of exposure, the biological activity of the cells was assessed for all methodologies using cytotoxicity (Alamar blue, LDH), inflammation (IL-1b, IL-6, IL-8, TNF-α levels in culture medium (ELISA)), and oxidative stress assays (DCF assay).

### Deposited dose assessment

In vivo, the mass of each NM instilled into the lungs was measured by ICP-MS. The nominal doses (5; 50; 500 μg/animal) were corrected to 4; 40 and 400 μg/animal according to dosage results (Additional file 1: Figure S5). According to the lung alveolar surface (4000 cm^2^) or the number of alveolar macrophages (25 million), this corresponds to theoretical deposited doses in lungs of around 0.1; 0.01; 0.001 μg/cm^2^ or 16; 1.6; 0.16 μg/10^6^ macrophages, respectively.

In vitro, the real mass deposited on the cells was either assessed by ICP-MS dosage (for ALI exposures) or estimated (in submerged conditions) using the in vitro sedimentation diffusion and dosimetry model (ISDD) [58], after measuring the hydrodynamic diameter by dynamic light scattering and the effective density of the agglomerates following the Volumetric Centrifugation Method (VCM) [59]. The detailed material and methods used in vitro and all the deposition data are available in the following paper [13]. Deposited masses on cells in vitro are also presented in the Additional files section of the present manuscript (Additional file 1: Tables S1 and S6). The final measured or calculated doses tested were around 0,1; 1; 3 μg/cm^2^ at the ALI (for 3 h of maintained deposition + 21 h without deposition in the incubator), 1; 3; 10 μg/cm^2^ in submerged in inserts (for 3 h of maintained deposition + 21 h without deposition in the incubator) and 1, 3, 10, 20 μg/cm^2^ in submerged in plates (24 h of maintained deposition).

### Determination of critical dose intervals using benchmark dose-response modeling

All the in vivo and in vitro data were analyzed using the benchmark dose-response modeling software PROAST (RIVM, Bilthoven, The Netherlands). The PROAST software selects the optimal data fitting model from an exponential family of models. Briefly, for each cytokine and each exposure method used (in vivo, ALI (3 h + 21 h), submerged in inserts (3 h + 21 h), submerged in plates (24 h)), we determined the critical effect dose corresponding to a 20% increase of pro-inflammatory mediator levels compared to non-exposed controls and the benchmark dose lower confidence limit (BMDL) and the benchmark dose upper confidence limit (BMDU) of the interval for a 90% confidence. For each exposure method used and each NM, we then calculated the median value of the BMDL and the median value of the BMDU of the four pro-inflammatory mediators (IL-1β, IL-6, IL-8/KC-GRO, TNF-α), to determine a median dose interval for general pro-inflammatory response. We decided to calculate the median dose interval of the four cytokines because similar results could be observed when pooling the results of the four cytokines and when comparing dose intervals of each cytokine one by one. We believe that our comparisons were easier to interpret in our study when using a general pro-inflammatory response. We choose a critical effect of 20% based on the magnitude of effect in several notable studies [16, 60, 61].

## Additional files

Additional file 1: **Figure S1.** Levels of pro-inflammatory mediators in cell supernatants in vitro (adapted from [13]). **Figure S2.** Levels of proteins, LDH (cytotoxicity) and intracellular ROS (oxidative stress) in BALF. **Figure S3.** Examples of critical effect doses (CED) and dose intervals (CEDL/BMDL and CEDU/BMDU) determined using benchmark dose response modeling. **Figure S4.** Size distribution of the NMs in the suspensions used to expose rats. **Figure S5.** Initial lung burden in vivo assessed by ICP-MS 3 h after instillation (*n* = 2). **Table S1.** Doses deposited in vitro in submerged conditions in function of nominal concentrations in suspensions (First published in [13]). **Table S2.** LOAELs (in μg/cm^2^) determined in vitro with the pro-inflammatory effects for each exposure method used (First published in [13]). **Table S3.** Dose intervals (in μg/10^6^ macrophages) determined for each NM and each methodology. **Table S4.** Dose intervals normalized by primary surface areas (in cm^2^/10^6^macrophages) for each NM and methodology. **Table S5.** Dose intervals normalized by agglomerate surface areas (in cm^2^/10^6^ macrophages) for each NM and methodology. **Table S6.** Characterization of mass deposited in vitro on cells after 3 h exposure at the ALI to aerosols of NMs (Adapted from [13]). (DOCX 831 kb)

## Abbreviations

ALI: Air-Liquid Interface
BALF: BronchoAlveolar Lavage Fluids
BET: Brunauer–Emmett–Teller
BMDL: Benchmark Dose Lower confidence limit
BMDU: Benchmark Dose Upper confidence limit
DLS: Dynamic Light Scattering
DMSO: Dimethyl Sulfoxide
ICP-MS: Inductively Coupled Plasma - Mass Spectrometry
ISDD: In vitro Sedimentation Diffusion and Dosimetry
JRC: Joint Research center
LDH: Lactate DeHydrogenase
LOAEL: Lowest Observed Adverse Effect Level
MGG: May-Grunwald Giemsa
NM(s): NanoMaterial(s)
NOAEL: No Observed Adverse Effect Level
PMA: Phorbol Myristate Acetate
ROS: Reactive Oxygen Species
SD: Standard Deviation
VCM: Volumetric Centrifugation Method
