# Supplementary material for: Predicting the in vivo pulmonary toxicity induced by acute exposure to poorly soluble nanomaterials by using advanced in vitro methods
Source: Part Fibre Toxicol. 2018 Jun 4;15:25. doi: 10.1186/s12989-018-0260-6 (PMC5987386; doi:10.1186/s12989-018-0260-6)
Supplement: Supplementary file 1 — Figure S1. Levels of pro-inflammatory mediators in cell supernatants in vitro (adapted from [13]). Figure S2. Levels of proteins, LDH (cytotoxicity) and intracellular ROS (oxidative stress) in BALF. Figure S3. Examples of critical effect doses (CED) and dose intervals (CEDL/BMDL and CEDU/BMDU) determined using benchmark dose response modeling. Figure S4. Size distribution of the NMs in the suspensions used to expose rats. Figure S5. Initial lung burden in vivo assessed by ICP-MS 3 h after instillation (n = 2). Table S1. Doses deposited in vitro in submerged conditions in function of nominal concentrations in suspensions (First published in [13]). Table S2. LOAELs (in μg/cm2) determined in vitro with the pro-inflammatory effects for each exposure method used (First published in [13]). Table S3. Dose intervals (in μg/106 macrophages) determined for each NM and each methodology. Table S4. Dose intervals normalized by primary surface areas (in cm2/106macrophages) for each NM and methodology. Table S5. Dose intervals normalized by agglomerate surface areas (in cm2/106 macrophages) for each NM and methodology. Table S6. Characterization of mass deposited in vitro on cells after 3 h exposure at the ALI to aerosols of NMs (Adapted from [13]). (DOCX 831 kb) [file 12989_2018_260_MOESM1_ESM.docx]

**Supplementary material**

**Figures**

**Figure S1.** Levels of pro-inflammatory mediators in cell supernatants *in vitro* (adapted from [[1](#_ENREF_1)3]).

**Figure S2.** Levels of proteins, LDH (cytotoxicity) and intracellular ROS (oxidative stress) in BALF.

**Figure S3.** Examples of critical effect doses (CED) and dose intervals (CEDL/BMDL and CEDU/BMDU) determined using benchmark dose response modeling.

**Figure S4.** Size distribution of the NMs in water suspensions used to expose rats.

**Figure S5.** Initial lung burden *in vivo* assessed by ICP-MS 3h after instillation (n=2).

**Tables**

**Table S1.** Doses deposited *in vitro* in submerged conditions in function of nominal concentrations in suspensions (First published in [[1](#_ENREF_1)3] ).

**Table S2.** LOAELs (in µg/cm²) determined *in vitro* with the pro-inflammatory effects for each exposure method used (First published in [[1](#_ENREF_1)3]).

**Table S3.** Dose intervals (in µg/10^6^ macrophages) determined for each NM and each methodology.

**Table S4.** Dose intervals normalized by primary surface areas (in cm²/10^6^ macrophages) for each NM and each methodology.

**Table S5.** Dose intervals normalized by agglomerate surface areas (in cm²/10^6^ macrophages) for each NM and methodology.

**Table S6.** Characterization of mass deposited *in vitro* on cells after 3h exposure at the ALI to aerosols of NMs (Adapted from [[1](#_ENREF_1)3]).

**Tables of results**

**Table S7.** Total number of cells in bronchoalveolar lavage fluids *in vivo.*

**Table S8.** Number of macrophages in bronchoalveolar lavage fluids *in vivo.*

**Table S9.** Number of neutrophils in bronchoalveolar lavage fluids *in vivo.*

**Table S10.** IL-1β secretion in bronchoalveolar lavage fluids *in vivo.*

**Table S11.** IL-6 secretion in bronchoalveolar lavage fluids *in vivo.*

**Table S12.** KC-GRO secretion in bronchoalveolar lavage fluids *in vivo.*

**Table S13.** TNF-α secretion in bronchoalveolar lavage fluids *in vivo.*

**References**

**Figures**


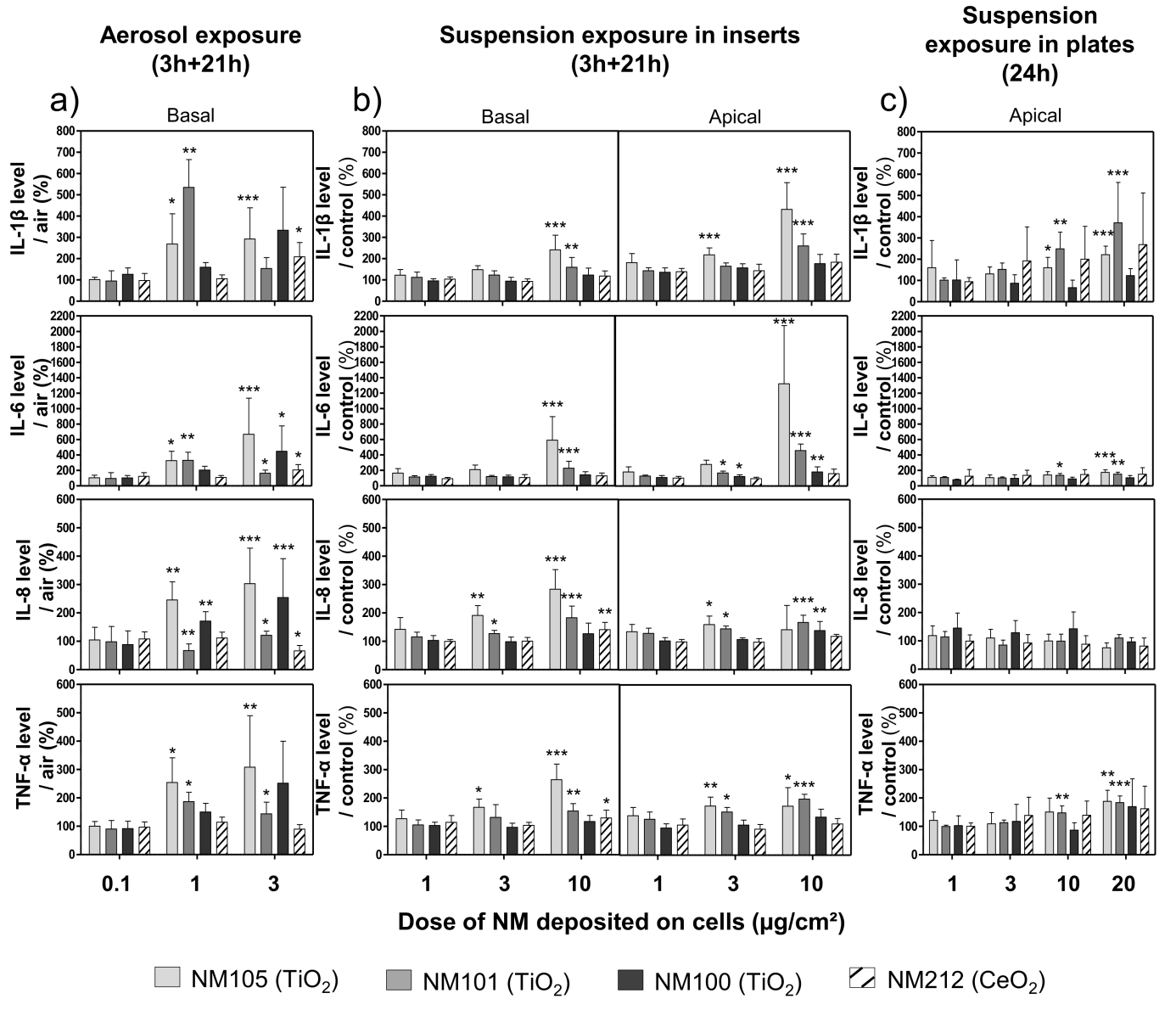


**Figure S1. Levels of pro-inflammatory mediators in cell supernatants *in vitro* (adapted from [**[**1**](#_ENREF_1)**3]).**

**Figure S2. Levels of proteins, LDH (cytotoxicity) and intracellular ROS (oxidative stress) in BALF.**

**Figure S3. Examples of critical effect doses (CED) and dose intervals (CEDL and CEDU) determined using benchmark dose response modeling.**

**Figure S4. Size distribution of the NMs in water suspensions used to expose rats.**

******

**Figure S5. Initial lung burden *in vivo* assessed by ICP-MS 3h after instillation (n=2).**

**Tables**

**Table S1. Doses deposited *in vitro* in submerged conditions in function of nominal concentrations in suspensions (First published in [**[**1**](#_ENREF_1)**3]).**

|  |  | **24h deposition in plates** | | | | **3h deposition in inserts** | | |
| --- | --- | --- | --- | --- | --- | --- | --- | --- |
| TiO_2_ NM105 | Nominal dose (µg/mL) | 10 | 50 | 100 | 200 | 54.5 | 163.5 | 544.9 |
|  | Nominal dose (µg/cm²) | 2.5 | 12.5 | 25 | 50 | 11.7 | 35.0 | 116.7 |
|  | Estimated dose using the ISDD model (µg/cm²) | **0.7** | **3.6** | **7.1** | **14.3** | **1** | **3** | **10** |
| TiO_2_ NM101 | Nominal dose (µg/mL) | 4 | 10 | 50 | 100 | 23.4 | 70.1 | 233.5 |
|  | Nominal dose (µg/cm²) | 1 | 2.5 | 12.5 | 25 | 5.0 | 15.0 | 50.0 |
|  | Estimated dose using the ISDD model (µg/cm²) | **1.0** | **2.5** | **12.5** | **25.0** | **1** | **3** | **10** |
| TiO_2_ NM100 | Nominal dose (µg/mL) | 4 | 10 | 50 | 100 | 34.3 | 102.9 | 343.1 |
|  | Nominal dose (µg/cm²) | 1 | 2.5 | 12.5 | 25 | 7.3 | 22.0 | 73.5 |
|  | Estimated dose using the ISDD model (µg/cm²) | **0.7** | **1.8** | **8.8** | **17.5** | **1** | **3** | **10** |
| CeO_2_ NM212 | Nominal dose (µg/mL) | 10 | 50 | 100 | 200 | 42.5 | 127.4 | 424.5 |
|  | Nominal dose (µg/cm²) | 2.5 | 12.5 | 25 | 50 | 9.1 | 27.3 | 90.9 |
|  | Estimated dose using the ISDD model (µg/cm²) | **0.9** | **4.7** | **9.5** | **18.9** | **1** | **3** | **10** |
| **Tested doses about (µg/cm²)** | | **1** | **3** | **10** | **20** | **1** | **3** | **10** |

**Table S2. LOAELs (in µg/cm²) determined *in vitro* with the pro-inflammatory effects for each exposure method used (First published in [**[**1**](#_ENREF_1)**3]).**

|  | IL-1β | | | IL-6 | | | IL-8 | | | TNF-α | | |
| --- | --- | --- | --- | --- | --- | --- | --- | --- | --- | --- | --- | --- |
|  | ALI^a^ | Subm insert^b^ | Subm  plate^c^ | ALI^a^ | Subm insert^b^ | Subm  plate^c^ | ALI^a^ | Subm insert^b^ | Subm  plate^c^ | ALI^a^ | Subm insert^b^ | Subm  plate^c^ |
| NM105 | 1 | 3 | 10 | 1 | 1 | 3 | 10 | 1 | 1 | 3 | 10 | 1 |
| NM101 | 1 | 10 | 10 | 1 | 1 | 10 | 10 | 1 | 1 | 10 | 10 | 1 |
| NM100 | Ø | Ø | Ø | 3 | Ø | Ø | Ø | 3 | Ø | Ø | Ø | 3 |
| NM212 | 3 | Ø | Ø | 3 | 3 | Ø | Ø | 3 | 3 | Ø | Ø | 3 |

^a^Exposure at the air-liquid interface (ALI) in insert for 24h. Final deposited within 3h.

^b^Exposure in submerged conditions in inserts for 24h. Final dose deposited within 3h.

^c^Exposure in submerged conditions in plates. Final dose deposited within 24h.

Ø No effects measured at the tested doses

**Table S3. Dose intervals (in µg/10^6^ macrophages) determined for each NM and each methodology.**

|  | Cytokines | NM105 | | | NM101 | | | NM100 | | | NM212 | | |
| --- | --- | --- | --- | --- | --- | --- | --- | --- | --- | --- | --- | --- | --- |
|  |  | Dose interval | | | Dose interval | | | Dose interval | | | Dose interval | | |
|  |  | BMDL | BMDU | Median | BMDL | BMDU | Median | BMDL | BMDU | Median | BMDL | BMDU | Median |
| *In vitro*,  suspension  (24h) | IL-1β | 3.83 | 475.94 | 185.23  -  447.98 | 21.19 | 114.01 | 112.93  -  254.43 | 427.32 | 1303.4 | 438.55  -  1047.6 | 2.5197 | 410.28 | 17.21  -  566.71 |
|  | IL-6 | 180.05 | 444.26 |  | 194.47 | 418.63 |  | ND | ND |  | ND | ND |  |
|  | IL-8 | ND | ND |  | ND | ND |  | ND | ND |  | ND | ND |  |
|  | TNF- α | 190.4 | 448.0 |  | 112.9 | 254.4 |  | 449.8 | 791.8 |  | 31.9 | 723.13 |  |
| *In vit*ro,  suspension  (3h + 21h) | IL-1β | 7.22 | 37.63 | 4.30  -  25.20 | 10.085 | 110.38 | 13.37  -  103.42 | 57.994 | 888.95 | 57.99  -  929.20 | 57.99 | 915.3 | 56.21  -  876.70 |
|  | IL-6 | 3.38 | 22.65 |  | 21.43 | 136.47 |  | ND | ND |  | 38.48 | 839.14 |  |
|  | IL-8 | 1.86 | 18.58 |  | 14.041 | 62.361 |  | 55.996 | 969.44 |  | 57.181 | 161.06 |  |
|  | TNF- α | 5.2 | 27.8 |  | 12.7 | 96.5 |  | 63.9 | 883.0 |  | 55.23 | 914.25 |  |
| ALI  (3h + 21h) | IL-1β | 0.85 | 13.36 | 1.01  -  13.64 | 1.02 | 12.41 | 1.64  -  13.43 | 0.092 | 15.14 | 0.75  -  15.05 | 10.47 | 43.57 | 14.69  -  45.28 |
|  | IL-6 | 0.61 | 13.53 |  | 1.48 | 12.86 |  | 0.195 | 14.647 |  | 14.69 | 45.28 |  |
|  | IL-8 | 1.31 | 13.78 |  | 18.89 | 198.27 |  | 1.30 | 14.96 |  | ND | ND |  |
|  | TNF- α | 1.17 | 13.75 |  | 1.81 | 14.00 |  | 3.96 | 16.12 |  | ND | ND |  |
| *In vivo* | IL-1β | 0.18 | 10.71 | 0.11  -  12.06 | ND | ND | 0.35  -  13.45 | ND | ND | ND | ND | ND | 0.00  -  11.85 |
|  | IL-6 | 0.11 | 22.26 |  | 0.71 | 13.77 |  | ND | ND |  | 0.00 | 3.10 |  |
|  | IL-8 | 0.03 | 13.40 |  | ND | ND |  | ND | ND |  | 0.52 | 13.07 |  |
|  | TNF- α | 0.12 | 1.46 |  | 0.00 | 13.14 |  | ND | ND |  | 0.00 | 11.85 |  |

BMDL: Benchmark Dose Lower confidence limit

BMDU: Benchmark Dose Upper confidence limit

Median: Median BMDL and BMDU values calculated by pooling the four cytokines, to have a dose-interval for a general pro-inflammatory response

**Table S4. Dose intervals normalized by primary surface areas (in cm²/10^6^ macrophages) for each NM and methodology.**

|  | Cytokines | NM105 | | | NM101 | | | NM100 | | | NM212 | | |
| --- | --- | --- | --- | --- | --- | --- | --- | --- | --- | --- | --- | --- | --- |
|  |  | Dose interval | | | Dose interval | | | Dose interval | | | Dose interval | | |
|  |  | BMDL | BMDU | Median | BMDL | BMDU | Median | BMDL | BMDU | Median | BMDL | BMDU | Median |
| *In vitro,*  suspension  (24h) | IL-1β | 1.77 | 219.41 | 85.39  -  206.52 | 66.96 | 360.27 | 356.86  -  804.00 | 42.73 | 130.34 | 43.86  -  104.76 | 0.68 | 110.78 | 4.65  -  153.01 |
|  | IL-6 | 83.00 | 204.80 |  | 614.53 | 1322.87 |  | ND | ND |  | ND | ND |  |
|  | IL-8 | ND | ND |  | ND | ND |  | ND | ND |  | ND | ND |  |
|  | TNF- α | 87.77 | 206.53 |  | 356.76 | 803.90 |  | 44.98 | 79.18 |  | 8.61 | 195.25 |  |
| *In vitro,*  suspension  (3h + 21h) | IL-1β | 3.33 | 17.35 | 1.98  -  11.62 | 31.87 | 348.80 | 42.26  -  326.82 | 5.80 | 88.90 | 5.80  -  92.92 | 15.66 | 247.13 | 15.18  -  236.71 |
|  | IL-6 | 1.56 | 10.44 |  | 67.72 | 431.25 |  | ND | ND |  | 10.39 | 226.57 |  |
|  | IL-8 | 0.86 | 8.57 |  | 44.37 | 197.06 |  | 5.60 | 96.94 |  | 15.44 | 43.49 |  |
|  | TNF- α | 2.40 | 12.82 |  | 40.13 | 304.94 |  | 6.39 | 88.30 |  | 14.91 | 246.85 |  |
| ALI  (3h + 21h) | IL-1β | 0.39 | 6.16 | 0.47  -  6.29 | 3.22 | 39.22 | 5.20  -  42.44 | 0.01 | 1.51 | 0.07  -  1.51 | 2.83 | 11.76 | 3.97  -  12.23 |
|  | IL-6 | 0.28 | 6.24 |  | 4.68 | 40.64 |  | 0.02 | 1.46 |  | 3.97 | 12.23 |  |
|  | IL-8 | 0.60 | 6.35 |  | 59.69 | 626.53 |  | 0.13 | 1.50 |  | ND | ND |  |
|  | TNF- α | 0.54 | 6.34 |  | 5.72 | 44.24 |  | 0.40 | 1.61 |  | ND | ND |  |
| *In vivo* | IL-1β | 0.08 | 4.94 | 0.05  -  5.56 | ND | ND | 1.12  -  42.51 | ND | ND | ND | ND | ND | 0.00  -  3.20 |
|  | IL-6 | 0.05 | 10.26 |  | 2.24 | 43.51 |  | ND | ND |  | 0.00 | 0.84 |  |
|  | IL-8 | 0.01 | 6.18 |  | ND | ND |  | ND | ND |  | 0.14 | 3.53 |  |
|  | TNF- α | 0.06 | 0.67 |  | 0.00 | 41.52 |  | ND | ND |  | 0.00 | 3.20 |  |

BMDL: Benchmark Dose Lower confidence limit

BMDU: Benchmark Dose Upper confidence limit

Median: Median BMDL and BMDU values calculated by pooling the four cytokines, to have a dose-interval for a general pro-inflammatory response

**Table S5. Dose intervals normalized by agglomerate surface areas (in cm²/10^6^ macrophages) for each NM and methodology.**

|  | Cytokines | NM105 | | | NM101 | | | NM100 | | | NM212 | | |
| --- | --- | --- | --- | --- | --- | --- | --- | --- | --- | --- | --- | --- | --- |
|  |  | Dose interval | | | Dose interval | | | Dose interval | | | Dose interval | | |
|  |  | BMDL | BMDU | Median | BMDL | BMDU | Median | BMDL | BMDU | Median | BMDL | BMDU | Median |
| *In vitro,*  suspension  (24h) | IL-1β | 0.52 | 64.44 | 25.08  -  60.65 | 1.43 | 7.69 | 7.62  -  17.16 | 50.36 | 153.62 | 51.69  -  123.47 | 0.32 | 51.40 | 2.16  -  70.99 |
|  | IL-6 | 24.38 | 60.15 |  | 13.12 | 28.23 |  | ND | ND |  | ND | ND |  |
|  | IL-8 | ND | ND |  | ND | ND |  | ND | ND |  | ND | ND |  |
|  | TNF- α | 25.78 | 60.65 |  | 7.61 | 17.16 |  | 53.01 | 93.32 |  | 4.00 | 90.59 |  |
| *In vitro,*  suspension  (3h + 21h) | IL-1β | 0.98 | 5.09 | 0.58  -  3.41 | 0.68 | 7.44 | 0.90  -  6.97 | 6.84 | 104.77 | 6.84  -  109.51 | 7.26 | 114.66 | 7.04  -  109.82 |
|  | IL-6 | 0.46 | 3.07 |  | 1.45 | 9.20 |  | ND | ND |  | 4.82 | 105.12 |  |
|  | IL-8 | 0.25 | 2.52 |  | 0.95 | 4.21 |  | 6.60 | 114.26 |  | 7.16 | 20.18 |  |
|  | TNF- α | 0.70 | 3.76 |  | 0.86 | 6.51 |  | 7.53 | 104.07 |  | 6.92 | 114.53 |  |
| ALI  (3h + 21h) | IL-1β | 0.30 | 4.77 | 0.36  -  4.87 | 0.85 | 10.34 | 1.37  -  11.19 | 0.03 | 4.73 | 0.23  -  4.70 | 2.86 | 11.88 | 4.01  -  12.35 |
|  | IL-6 | 0.22 | 4.83 |  | 1.23 | 10.72 |  | 0.06 | 4.58 |  | 4.01 | 12.35 |  |
|  | IL-8 | 0.47 | 4.92 |  | 15.74 | 165.23 |  | 0.41 | 4.68 |  | ND | ND |  |
|  | TNF- α | 0.42 | 4.91 |  | 1.51 | 11.67 |  | 1.24 | 5.04 |  | ND | ND |  |
| *In vivo* | IL-1β | 0.02 | 1.45 | 0.02  -  1.63 | ND | ND | 0.02  -  0.91 | ND | ND | ND | ND | ND | 0.00  -  1.48 |
|  | IL-6 | 0.01 | 3.01 |  | 0.05 | 0.93 |  | ND | ND |  | 0.00 | 0.39 |  |
|  | IL-8 | 0.00 | 1.81 |  | ND | ND |  | ND | ND |  | 0.07 | 1.64 |  |
|  | TNF- α | 0.02 | 0.20 |  | 0.00 | 0.89 |  | ND | ND |  | 0.00 | 1.48 |  |

**Table S6. Characterization of mass deposited *in vitro* on cells after 3h exposure at the ALI to aerosols of NMs (Adapted from [**[**1**](#_ENREF_1)**3]).**

| Nebulizer (PALAS, AGK 2000) | TiO_2_ NM105 | | | TiO_2_ NM101 | | | | TiO_2_ NM100 | | | CeO_2_ NM212 | | |  |
| --- | --- | --- | --- | --- | --- | --- | --- | --- | --- | --- | --- | --- | --- | --- |
| Suspension concentration (g/L) | 1 | 5 | 10 | | 1 | 5 | 10 | 1 | 5 | 10 | 1 | 5 | 10 | |
| Deposited mass*^d^*(µg/cm^2^ in 3h) (n=4-7) (ICP-MS) | 0.06±  0.01 | 0.66±  0.12 | 2.68  ±  0.6 | | 0.22  ±  0.03 | 1.51  ±  0.11 | 3.15  ±  0.44 | 0.11  ±  0.01 | 0.50  ±  0.12 | 2.85  ±  0.30 | 0.22  ±  0.02 | 1.54  ±  0.08 | 3.26  ±  0.68 | |

**Tables of results**

**Table S7. Total number of cells in bronchoalveolar lavage fluids *in vivo.***

| Total cells  (x 10^6^) | **NM105** | | | **NM101** | | | **NM100** | | | **NM212** | | |
| --- | --- | --- | --- | --- | --- | --- | --- | --- | --- | --- | --- | --- |
| Dose (µg) | Mean | SD | n | Mean | SD | n | Mean | SD | n | Mean | SD | n |
| 0 | 8,116667 | 2,469143 | 6 | 8,320 | 1,104681 | 6 | 8,320 | 1,104681 | 6 | 6,758333 | 0,8873649 | 6 |
| 5 | 11,94167 | 7,829394 | 6 | 7,606667 | 1,515449 | 6 | 7,041667 | 1,81863 | 6 | 7,378333 | 2,405497 | 6 |
| 50 | 10,125 | 3,014424 | 6 | 8,580 | 2,256333 | 6 | 9,500 | 3,018609 | 6 | 7,758334 | 1,546744 | 6 |
| 500 | 13,40833 | 7,89116 | 6 | 6,480 | 1,718232 | 6 | 8,866667 | 2,73636 | 6 | 6,595 | 1,546205 | 6 |

**Table S8. Number of macrophages in bronchoalveolar lavage fluids *in vivo.***

| Macrophages  (x 10^6^) | **NM105** | | | **NM101** | | | **NM100** | | | **NM212** | | |
| --- | --- | --- | --- | --- | --- | --- | --- | --- | --- | --- | --- | --- |
| Dose (µg) | Mean | SD | n | Mean | SD | n | Mean | SD | n | Mean | SD | n |
| 0 | 7,906667 | 2,405815 | 6 | 8,088333 | 1,261942 | 6 | 8,088333 | 1,261942 | 6 | 6,400 | 0,7920605 | 6 |
| 5 | 11,80167 | 7,818852 | 6 | 6,755 | 1,747624 | 6 | 7,083333 | 1,690535 | 6 | 7,245 | 2,231123 | 6 |
| 50 | 9,778334 | 2,919284 | 6 | 9,286667 | 3,087139 | 6 | 8,116667 | 2,073438 | 6 | 7,330 | 1,470687 | 6 |
| 500 | 12,45333 | 7,389264 | 6 | 8,585 | 2,852155 | 6 | 5,630 | 1,935407 | 6 | 5,946667 | 1,534675 | 6 |

**Table S9. Number of neutrophils in bronchoalveolar lavage fluids *in vivo.***

| Neutrophils  (x 10^6^) | **NM105** | | | **NM101** | | | **NM100** | | | **NM212** | | |
| --- | --- | --- | --- | --- | --- | --- | --- | --- | --- | --- | --- | --- |
| Dose (µg) | Mean | SD | n | Mean | SD | n | Mean | SD | n | Mean | SD | n |
| 0 | 2,533333 | 1,834848 | 6 | 0,650 | 0,5504543 | 6 | 0,650 | 0,5504543 | 6 | 5,000 | 6,131884 | 6 |
| 5 | 1,383333 | 1,937438 | 6 | 6,950 | 7,524294 | 6 | 3,550 | 3,894997 | 6 | 1,333333 | 2,160247 | 6 |
| 50 | 3,100 | 2,565151 | 6 | 4,850 | 3,924666 | 6 | 2,250 | 3,318283 | 6 | 5,166667 | 2,316607 | 6 |
| 500 | 6,766666 | 3,631896 | 6 | 13,33333 | 12,72143 | 6 | 3,633333 | 2,063654 | 6 | 9,833333 | 4,833908 | 6 |

**Table S10. IL-1β secretion in bronchoalveolar lavage fluids *in vivo.***

| IL-1β (pg/mL) | **NM105** | | | **NM101** | | | **NM100** | | | **NM212** | | |
| --- | --- | --- | --- | --- | --- | --- | --- | --- | --- | --- | --- | --- |
| Dose (µg) | Mean | SD | n | Mean | SD | n | Mean | SD | n | Mean | SD | n |
| 0 | 8,884489 | 1,849256 | 6 | 9,138938 | 4,634266 | 6 | 9,138938 | 4,634266 | 6 | 5,91275 | 2,010868 | 6 |
| 5 | 8,277813 | 2,307245 | 6 | 11,53626 | 5,389348 | 6 | 10,98688 | 6,011103 | 6 | 9,572947 | 4,58724 | 6 |
| 50 | 10,45812 | 2,844312 | 6 | 9,932984 | 3,694911 | 6 | 6,774626 | 2,236386 | 6 | 6,11853 | 0,7779508 | 6 |
| 500 | 11,85874 | 2,137961 | 6 | 13,254 | 11,96707 | 6 | 6,882884 | 2,226868 | 6 | 9,275265 | 2,540267 | 6 |

**Table S11. IL-6 secretion in bronchoalveolar lavage fluids *in vivo.***

| IL-6  (pg/mL) | **NM105** | | | **NM101** | | | **NM100** | | | **NM212** | | |
| --- | --- | --- | --- | --- | --- | --- | --- | --- | --- | --- | --- | --- |
| Dose (µg) | Mean | SD | n | Mean | SD | n | Mean | SD | n | Mean | SD | n |
| 0 | 641,9868 | 1090,115 | 6 | 201,9327 | 409,2815 | 6 | 201,9327 | 409,2815 | 6 | 247,1255 | 251,5747 | 6 |
| 5 | 83,94656 | 123,9007 | 6 | 1561,140 | 2454,902 | 6 | 1728,992 | 2213,854 | 6 | 646,437 | 1215,112 | 6 |
| 50 | 299,6054 | 373,0139 | 6 | 102,3409 | 130,1647 | 6 | 247,705 | 340,396 | 6 | 575,3152 | 452,0587 | 6 |
| 500 | 494,0656 | 372,196 | 6 | 2547,297 | 5762,785 | 6 | 124,1872 | 177,432 | 6 | 2057,351 | 1911,255 | 6 |

**Table S12. KC-GRO secretion in bronchoalveolar lavage fluids *in vivo.***

| KC-GRO  (pg/mL) | **NM105** | | | **NM101** | | | **NM100** | | | **NM212** | | |
| --- | --- | --- | --- | --- | --- | --- | --- | --- | --- | --- | --- | --- |
| Dose (µg) | Mean | SD | n | Mean | SD | n | Mean | SD | n | Mean | SD | n |
| 0 | 154,921 | 130,2589 | 6 | 436,0563 | 849,0498 | 6 | 436,0563 | 849,0498 | 6 | 612,267 | 769,132 | 6 |
| 5 | 117,6946 | 33,43017 | 6 | 505,3957 | 611,5626 | 6 | 525,504 | 449,3068 | 6 | 245,1932 | 185,7892 | 6 |
| 50 | 357,3673 | 484,7474 | 6 | 101,2313 | 68,70238 | 6 | 238,2058 | 235,6528 | 6 | 255,8444 | 96,38566 | 6 |
| 500 | 514,3612 | 597,0824 | 6 | 584,2678 | 709,4099 | 6 | 259,6591 | 309,2401 | 6 | 1358,297 | 933,4235 | 6 |

**Table S13. TNF-α secretion in bronchoalveolar lavage fluids *in vivo.***

| TNF-α  (pg/mL) | **NM105** | | | **NM101** | | | **NM100** | | | **NM212** | | |
| --- | --- | --- | --- | --- | --- | --- | --- | --- | --- | --- | --- | --- |
| Dose (µg) | Mean | SD | n | Mean | SD | n | Mean | SD | n | Mean | SD | n |
| 0 | 1,704346 | 0,8449346 | 6 | 1,903974 | 2,234239 | 6 | 1,903974 | 2,234239 | 6 | 2,425805 | 1,646873 | 6 |
| 5 | 1,420876 | 0,4093659 | 6 | 2,528478 | 2,363132 | 6 | 2,463016 | 1,448268 | 6 | 3,038258 | 2,296852 | 6 |
| 50 | 2,914879 | 0,9622893 | 6 | 2,734777 | 2,081956 | 6 | 1,390201 | 0,7100273 | 6 | 5,076797 | 5,844111 | 6 |
| 500 | 6,758596 | 2,074849 | 6 | 6,386044 | 4,192086 | 6 | 1,478584 | 0,8414167 | 6 | 8,041635 | 3,607172 | 6 |

**Reference**

13. Loret T, Peyret E, Dubreuil M, Aguerre-Chariol O, Bressot C, le Bihan O, et al. Air-liquid interface exposure to aerosols of poorly soluble nanomaterials induces different biological activation levels compared to exposure to suspensions. Part Fibre Toxicol. 2016;13(1):58.
